# A practical guide to methods controlling false discoveries in computational biology

**DOI:** 10.1186/s13059-019-1716-1

**Published:** 2019-06-04

**Authors:** Keegan Korthauer, Patrick K. Kimes, Claire Duvallet, Alejandro Reyes, Ayshwarya Subramanian, Mingxiang Teng, Chinmay Shukla, Eric J. Alm, Stephanie C. Hicks

**Affiliations:** 1 Department of Data Sciences, Dana-Farber Cancer Institute, 450 Brookline Avenue, Boston, 02215 USA; 2 Department of Biostatistics, Harvard T.H. Chan School of Public Health, 677 Huntington Avenue, Boston, 02215 USA; 3 Department of Biological Engineering, MIT, 77 Massachusetts Avenue, Cambridge, USA; 4 Center for Microbiome Informatics and Therapeutics, MIT, 77 Massachusetts Avenue, Cambridge, USA; 5 Broad Institute, 415 Main Street, Cambridge, USA; 6 Department of Biostatistics & Bioinformatics, Moffitt Cancer Center, 12902 Magnolia Drive, Tampa, 33612 USA; 7 Biological and Biomedical Sciences Program, Harvard University, Boston, USA; 8 Department of Biostatistics, Johns Hopkins Bloomberg School of Public Health, 615 N. Wolfe Street, Baltimore, 21205 USA

**Keywords:** Multiple hypothesis testing, False discovery rate, RNA-seq, ScRNA-seq, ChIP-seq, Microbiome, GWAS, Gene set analysis

## Abstract

**Background:**

In high-throughput studies, hundreds to millions of hypotheses are typically tested. Statistical methods that control the false discovery rate (FDR) have emerged as popular and powerful tools for error rate control. While classic FDR methods use only *p* values as input, more modern FDR methods have been shown to increase power by incorporating complementary information as informative covariates to prioritize, weight, and group hypotheses. However, there is currently no consensus on how the modern methods compare to one another. We investigate the accuracy, applicability, and ease of use of two classic and six modern FDR-controlling methods by performing a systematic benchmark comparison using simulation studies as well as six case studies in computational biology.

**Results:**

Methods that incorporate informative covariates are modestly more powerful than classic approaches, and do not underperform classic approaches, even when the covariate is completely uninformative. The majority of methods are successful at controlling the FDR, with the exception of two modern methods under certain settings. Furthermore, we find that the improvement of the modern FDR methods over the classic methods increases with the informativeness of the covariate, total number of hypothesis tests, and proportion of truly non-null hypotheses.

**Conclusions:**

Modern FDR methods that use an informative covariate provide advantages over classic FDR-controlling procedures, with the relative gain dependent on the application and informativeness of available covariates. We present our findings as a practical guide and provide recommendations to aid researchers in their choice of methods to correct for false discoveries.

**Electronic supplementary material:**

The online version of this article (10.1186/s13059-019-1716-1) contains supplementary material, which is available to authorized users.

## Background

When multiple hypotheses are simultaneously tested, an adjustment for the multiplicity of tests is often necessary to restrict the total number of false discoveries. The use of such adjustments for multiple testing has become standard in areas such as genomics [1, 2], neuroimaging [3], proteomics [4], psychology [5, 6], and economics [7]. Most classically, methods which control the family-wise error rate (FWER), or probability of at least one false discovery, have been developed and used to correct for multiple testing. These include the Bonferroni correction [8, 9] and other approaches [10–12]. Despite their popularity, FWER-controlling methods are often highly conservative, controlling the probability of any false positives (type I errors) at the cost of greatly reduced power to detect true positives. The trade-off of type I errors and power has become exacerbated in the analysis of data from high-throughput experiments, where the number of tests being considered can range from several thousand to several million.

**Fig. 1:**
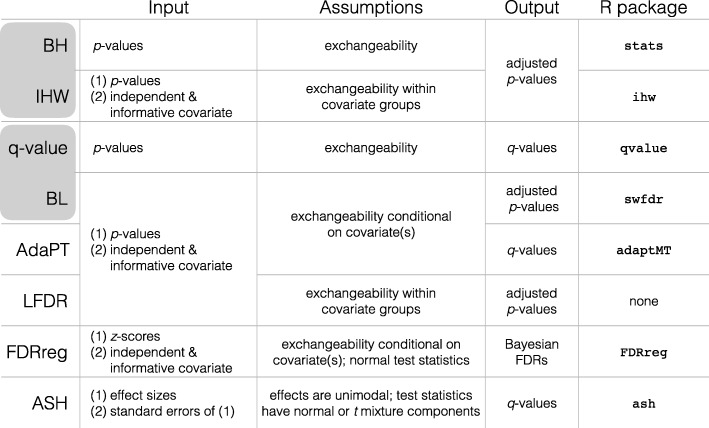
FDR-controlling methods included in the comparison. Inputs, assumptions, output, and availability (R package) of two classic [13, 14] and six modern [15–20] FDR-controlling methods. The outputs of the FDR-controlling methods vary, but they all can be used for the purpose of controlling the FDR. Pairs of classic and modern methods are highlighted in gray if the modern method is an extension of the classic method

The false discovery rate (FDR), or expected proportion of discoveries which are falsely rejected [13], was more recently proposed as an alternative metric to the FWER in multiple testing control. This metric has been shown to have greater power to detect true positives, while still controlling the proportion of type I errors at a specified level [13, 21]. In high-throughput biological experiments where investigators are willing to accept a small fraction of false positives to substantially increase the total number of discoveries, the FDR is often more appropriate and useful [22]. The Benjamini and Hochberg step-up procedure (BH) [13, 23] was the first method proposed to control the FDR. Soon afterwards, the *q*-value was introduced as a more powerful approach to controlling the FDR (Storey’s *q*-value) [14]. We refer to the BH procedure and Storey’s *q*-value as “classic” FDR-controlling methods (Fig. 1), because they can be easily computed with just a list of *p* values using robust software [24, 25] and are arguably still the most widely used and cited methods for controlling the FDR in practice.

While the BH procedure and Storey’s *q*-value often provide a substantial increase in discoveries over methods that control the FWER, they were developed under the assumption that all tests are exchangeable and, therefore, that the power to detect discoveries is equally likely among all tests. However, individual tests or groups of tests often differ in statistical properties, such as their level of precision, or underlying biology, which can lead to certain tests having greater power than others [15, 18]. For example, in a genome-wide association study (GWAS) meta-analysis where samples are pooled across studies, the loci-specific sample sizes can be informative of the differing signal-to-noise ratio across loci [16]. Additionally, in an expression quantitative trait loci (eQTL) study, tests between polymorphisms and genes in *cis* are known a priori to be more likely to be significant than those in *trans* [15].

Recently, a new class of methods that control the FDR (Fig. 1, Additional file 1: Table S1) has been proposed to exploit this variability across tests by combining the standard input (*p* values or test statistics) [13, 14, 26] with a second piece of information, referred to as an “informative covariate” [15–19, 27]. Intuitively, if a covariate is informative of each test’s power or prior probability of being non-null, it can be used to prioritize individual or groups of tests to increase the overall power of the experiment [15]. To guarantee FDR control, the covariate must also be independent of the *p* values under the null hypothesis. In a similar vein, other approaches have been proposed using two alternative pieces of information, namely effect sizes and their standard errors [20], to adaptively control the FDR. These modern FDR-controlling methods allow researchers to leverage additional information or metadata and are particularly well suited for biological studies.

However, due to their recent and concurrent development, comparisons between these modern FDR methods have been limited, and the demonstration of each method’s applicability and utility on real biological problems is highly variable. Furthermore, each method requires varying sets of input data and relies on differing sets of methodological assumptions. As a result, the answer to the simple question of which methods *can*, let alone *should*, be used for a particular analysis is often unclear.

To bridge the gap between methods and application, we performed a systematic benchmark comparison of two classic and six modern FDR-controlling methods. Specifically, we compared the classic BH approach [13] and Storey’s *q*-value [14] with several modern FDR-controlling methods, including the conditional local FDR (LFDR) [17], FDR regression (FDRreg) [19], independent hypothesis weighting (IHW) [15], adaptive shrinkage (ASH) [20], Boca and Leek’s FDR regression (BL) [16], and adaptive *p*-value thresholding (AdaPT) [18] (Fig. 1). Throughout, we use lowercase when referring to the specific, typically default, implementation of each method detailed in the “Methods” section. Both the theoretical and empirical null Empirical Bayes implementations of FDRreg were compared, referred to as “fdrreg-t” and “fdrreg-e,” respectively. AdaPT was compared using the default logistic-Gamma generalized linear model option and is referenced as “adapt-glm.” The *q* values returned by ASH were used for comparison and are referred to as “ashq.”

Of the modern FDR-controlling methods included in our comparison, IHW, BL, AdaPT, and LFDR can be applied generally to any multiple testing problem with *p* values and an informative covariate satisfying a minimal set of assumptions (Fig. 1, Additional file 1: Table S1). In contrast, FDRreg is restricted to multiple testing problems where normal test statistics, expressed as *z*-scores, are available. Mostly, unlike the other modern methods, ASH requires effect sizes and standard errors separately for normal or *t*-distributed test statistics and cannot be used with more general informative covariates. Furthermore, ASH requires that the true (unobserved) effect sizes across all tests are unimodal, i.e., that most non-null effect sizes are small and near zero. While this may be a reasonable assumption in settings where most non-null effects are believed to be small and larger effects are rare, it might not necessarily be true for all datasets and applications. While it is not possible to confirm whether the assumption is true, it is simple to check whether the assumption is blatantly violated, i.e., if the distribution of all observed effect sizes shows clear multimodality.

While both the BH procedure and Storey’s *q*-value serve as reference points for evaluating the modern FDR-controlling methods, in Fig. 1 (and in Additional file 1: Table S1), we highlight two pairs of modern and classic methods with a special relationship: IHW with the BH procedure and BL with Storey’s *q*-value. In the case that a completely uninformative covariate is used, these modern methods have the attractive property of reducing to their classic counterparts, subject to some estimation error. Therefore, when instructive, direct comparisons are also made between IHW and the BH procedure, and similarly between BL and Storey’s *q*-value.

In this paper, we first evaluate the performance and validity of these methods using simulated data and in silico RNA-seq spike-in datasets. Then, we investigate the applicability of these methods to multiple testing problems in computational biology through a series of six case studies, including differential expression testing in bulk RNA-seq, differential expression testing in single-cell RNA-seq, differential abundance testing and correlation analysis in 16S microbiome data, differential binding testing in ChIP-seq, genome-wide association testing, and gene set analysis. Combining these results with insights from our simulation studies and in silico experiments, we provide a key set of recommendations to aid investigators looking to take advantage of advances in multiple testing correction in future studies.

## Results

Although fdrreg-e was included in the benchmarking study, we exclude it from the presentation of the main results due to its unstable and inferior performance to its counterpart fdrreg-t. For detailed results including fdrreg-e, we refer the reader to Additional file 1.

### False discovery rate control

The specificity of the FDR-controlling methods was evaluated using three approaches. First, a series of RNA-seq differential expression studies were performed on yeast in silico spike-in datasets generated by randomly selecting 2 sets of 5 and 10 samples each from a dataset of 48 biological replicates in a single condition [29] and adding differential signal to a subset of genes to define “true positives.” This was carried out for a variety of settings of non-null effect size distributions, proportions of null hypotheses, and informativeness of covariates (Additional file 1: Table S2). Second, a similar differential expression study was performed using RNA-seq data simulated with the polyester R/Bioconductor package [28]. Finally, to explore a wider range of mutiple testing scenarios, an extensive simulation study was carried out across a range of test statistic distributions, non-null effect size distributions, proportions of null hypotheses, informative covariates, and numbers of tests (Additional file 1: Table S3).

All experiments and simulations were replicated 100 times. Performance metrics are reported as the mean and standard error across replications. In all analyses, covariate-aware modern FDR-controlling methods, including adapt-glm, bl, fdrreg-t, ihw, and lfdr, were run twice, once with an informative covariate and again with an uninformative random covariate.

While the notion of an informative covariate was loosely introduced above, for our *in silico* experiments and simulations, we concretely define “informative covariates” by introducing a dependence between the proportion of hypotheses that are null and the value of the covariate. A *strongly informative covariate* in our simulations is one where certain values of the covariate are highly enriched for truly non-null tests, and a *weakly informative covariate* is one where certain values are only moderately enriched for non-null tests. In contrast, an *uninformative covariate* is not enriched for null or non-null hypotheses for any values. We restrict the concepts of weakly and strongly informative covariates in our analysis to the dependence between the covariate and the null proportion described above. No other dependence is introduced between the covariate and the test statistics in our simulations and in silico experiments.

#### Modern methods do not always control the FDR

**Fig. 2:**
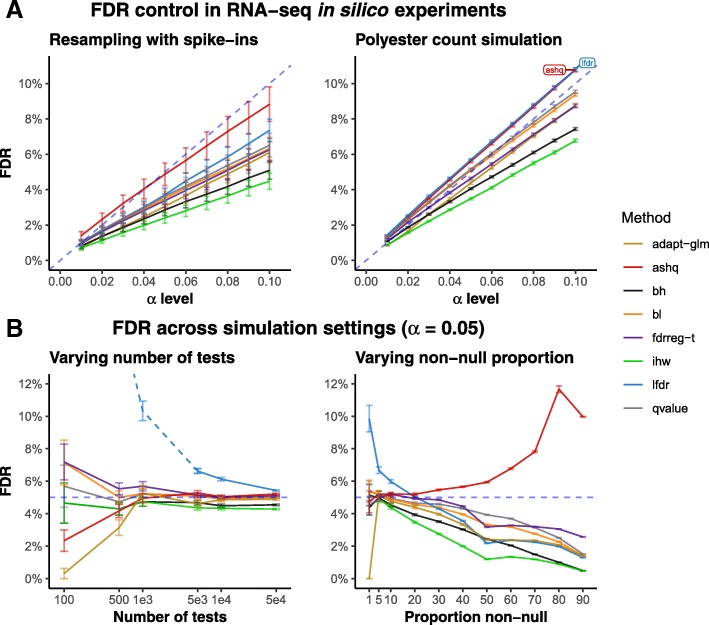
FDR control in *in silico* experiments and simulations. **a** Observed FDR (*y*-axis) for various *α*-level cutoffs (*x*-axis) in the yeast RNA-seq *in silico* resampling experiment with spiked-in differentially expressed genes (left panel) and the simulation of yeast RNA-seq counts using the polyester R/Bioconductor package [28]. **b** Observed FDR (*y*-axis) across simulation settings at *α*-level of 0.05. The left panel displays FDR for increasing numbers of hypothesis tests and the right panel displays FDR for increasing proportions of non-null hypotheses. Note that the LFDR method is displayed as a dotted line when the number of tests per bin falls below 200 (where the number of bins is fixed at 20), as fdrtools generates a warning in this case that the estimation may be unreliable

Across *in silico* experiments and simulation settings, we found that most methods adequately controlled the FDR in many situations. FDR control for a single setting of the yeast RNA-seq *in silico* experiments and Polyester count simulations is shown in Fig. 2a. In these experiments, 30% of genes were differentially expressed (DE) between the two groups of five samples each, with effect sizes sampled from a unimodal distribution and using a strongly informative covariate. Here, all methods controlled the FDR at the target *α*-levels between 0.01 and 0.10, with the exception of ashq and lfdr, which exhibited slightly inflated FDR in the polyester simulations. Some methods, most noticeably ihw, achieved lower FDR than others. The trade-off between FDR, power, and classification accuracy in the *in silico* experiments is summarized in Additional file 1: Figure S1.

The settings of the *in silico* experiments were varied to also consider a lower proportion of DE genes (7.5%), bimodal effect size distribution, and a weakly informative covariate in addition to the uninformative random covariate run with all covariate-aware methods (Additional file 1: Table S2). The FDR for covariate-aware methods was not sensitive to covariate informativeness, with nearly identical proportions of false discoveries using weakly and strongly informative covariates. However, we found that with the bimodal effect size distribution and smaller proportion of non-null hypotheses, a subset of methods including ashq, and lfdr, failed to control the FDR at the nominal FDR cutoff, leading to an inflated rate of false discoveries (Additional file 1: Figure S3).

Similar trends were observed across simulation studies, where conditions were varied analogous to the yeast experiments to consider a wider range of scenarios. While most methods were consistently conservative or achieved an accurate target FDR, a subset of methods clearly failed to control the FDR under certain settings.

#### lfdr and fdrreg-t do not control FDR with few tests

Since modern FDR-controlling methods must estimate the covariate dependence from the set of hypotheses, the effectiveness of these methods can depend on having a sufficiently large number of tests. We performed a series of simulations to assess the sensitivity of the covariate-aware methods to the total number of hypotheses. We observed that lfdr exhibited substantially inflated FDR when applied to 10,000 or fewer tests (Fig. 2b, left panel). This result could be due to our implementation of LFDR, which groups hypotheses into 20 groups regardless of the total number of tests, and suggests that the performance of lfdr improves when the numbers of tests per bin increases. We also observed that fdrreg-t showed slightly inflated FDR with 1000 or fewer tests.

#### lfdr and ashq do not control FDR for extreme proportions of non-null tests

The proportion of non-null tests is typically unknown but can vary dramatically between datasets. While most simulations were performed with 10% non-null tests, to cover a range of scenarios, a series of simulations covering non-null proportions between 0 and 95% were also considered. The yeast in silico experiments included settings of 0%, 7.5% and 30% non-null tests.

Most methods demonstrated the same general trend in simulation, where the FDR of most methods was controlled at the target *α*-level, and decreased as the proportion of non-null hypotheses increased (Fig. 2b, right panel). However, we also found that the ability of some methods to control FDR was sensitive to the proportion of tests that were non-null. Specifically, lfdr exhibited inflated FDR when the proportion of non-null tests was low (less than 20%). Likewise, ashq exhibited inflated FDR when the proportion of non-null tests was high (greater than 20%).

Similarly, ashq and lfdr failed to control FDR in the in silico yeast experiments when the proportion of non-nulls was 7.5% compared to 30% (Additional file 1: Figure S3). We also note that for a sample size of five per group, several methods exhibited inflated FDR in the extreme setting when the non-null proportion of hypotheses was 0%, where FDR reduces to FWER. However, although the proportion of replications with at least one false positive was greater than the target, the average proportion of tests rejected was very small (Additional file 2; see https://pkimes.github.io/benchmark-fdr-html/ [30]). Since the in silico experiments were generated by splitting biological replicates into two groups, it is possible that unmeasured biological differences exist between them.

### Power

In addition to FDR, we also evaluated sensitivity of the FDR-controlling methods using the same in silico experiment and simulation framework described above.

#### Modern methods are modestly more powerful

**Fig. 3:**
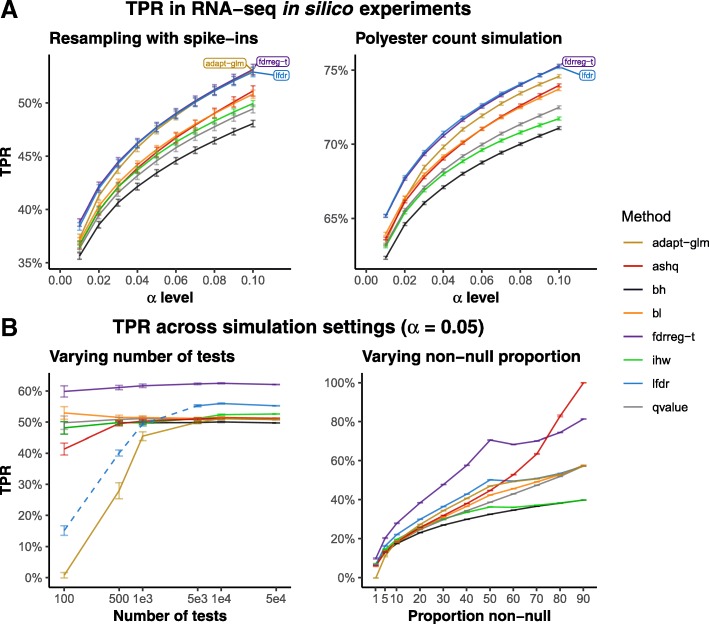
Power in in silico experiments and simulations. **a** True positive rate (*y*-axis) for increasing *α*-level cutoffs (*x*-axis) in the yeast RNA-seq in silico resampling experiment with spiked-in differentially expressed genes (left panel) and the simulation of yeast RNA-seq counts using the polyester R/Bioconductor package [28]. Similar plots in the style of [31] are displayed in Additional file 1: Figure S2. **b** True positive rate (*y*-axis) across simulation settings at *α*-level of 0.05. The left panel displays increasing numbers of hypothesis tests, and the right panel displays increasing proportions of non-null hypotheses. Note that the lfdr method is displayed as a dotted line when the number of tests per bin falls below 200 (where the number of bins is fixed at 20), as fdrtools generates a warning in this case that the estimation may be unreliable

We found that in general, modern FDR methods led to a modestly higher true positive rate (TPR), or power, in the yeast in silico RNA-seq experiments and polyester simulations (Fig. 3a, Additional file 1: Figure S2). This was also true when using a weakly informative rather than a strongly informative covariate (Additional file 1: Figure S3B). Much of the gain with modern methods, most apparent with lfdr and ashq, was found in genes with small to moderate effect sizes (Additional file 1: Figure S1D). While the majority of discoveries were common among all or most methods, there were several smaller sets of rejections that were unique to subsets of methods (Additional file 1: Figure S1E).

Again, higher power was similarly observed for most modern FDR methods over classic methods across simulation settings (Fig. 3b, Additional file 1: Figures S4, S5, S6, S7, S8). The increase in TPR was generally modest for all methods, as in the yeast experiments, with the exception of fdrreg-t which showed substantial improvement in TPR over modern methods in several simulation settings (Additional file 1: Figure S4B, D, and F).

#### Power of modern methods is sensitive to covariate informativeness

Comparing across yeast experiments using weakly and strongly informative covariates, we found that the TPR was higher for strongly informative covariates compared to weakly informative covariates (Additional file 1: Figure S3). To further quantify the impact of covariate informativeness, a series of simulations was performed using covariates of varying informativeness. A rough scale of 0–100 was used to describe the informativeness of the covariate, with larger values of informativeness corresponding to greater power of the covariate to distinguish null and non-null tests (Additional file 1: Figure S9). Echoing the results of the yeast experiments, the gain in TPR of covariate-aware methods over other methods also increased with informativeness in the simulation studies (Additional file 1: Figure S4B). This gain tended to be larger for some methods (fdrreg-t, lfdr, and adapt-glm) than for others (ihw and bl).

Additionally, simulations were performed across four different dependencies between the covariate and the null proportion. The covariates were named *step*, *cosine*, *sine*, and *cubic* for the shape of their dependence. We found that in general, the above results were relatively robust to the functional form of the dependence (Additional file 1: Figure S5A-C). However, the gain in TPR varied across informative covariates, with the smallest gains observed in the *step* covariate setting across all covariate-aware methods, likely attributable to the lower informativeness of the covariate relative to the other settings. The gain in TPR also varied more for some methods than others. In the *cosine* covariate, where the dependence between the covariate and null proportion was strongly non-monotone, bl showed no gain over the classic qvalue. As bl attempts to model the covariate-null proportion dependence using logistic regression, a monotone function, the method was unable to capture the true relationship. A small but noticeable increase in TPR was observed in the remaining settings, where the covariate dependence was monotone. In contrast, covariate-aware methods with more flexible modeling approaches either based on binning (ihw, lfdr) or spline expansion (fdrreg-t), were generally more consistent across covariates.

#### Including an uninformative covariate is not harmful

A reasonable concern, closely related to weakly and strongly informative covariates, is whether an uninformative covariate could mislead methods such as ihw, bl, fdrreg, lfdr, or adapt-glm. Across the settings of the yeast in silico experiments and simulations, we observed that with the use of a completely uninformative covariate, modern FDR methods generally had lower power (and higher FDR) than with an informative covariate (Additional file 1: Figures S5D-E, S6D-E, S7D-E, S10, and S11B). However, while modern FDR methods were modestly more powerful than classic approaches when using an informative covariate, they did not underperform classic approaches with a completely uninformative covariate (Additional file 1: Figure S4A-B).

A notable exception was adapt-glm, which suffered from lower power with the inclusion of a weakly informative covariate than with the uninformative covariate, likely due to overfitting (Additional file 1: Figures S4B and S5E). In estimating the dependence between the covariate and null proportion, adapt-glm includes a step of model selection. Based on a feedback from the method authors, we considered modifying the default adaplt-glm parameters by including a null dependence as one of the model choices, allowing the method to ignore the dependence when it cannot be properly estimated. When applied to the weakly informative *step* covariate setting, this resulted in improved performance with the method no longer suffering from lower power with the inclusion of the weakly informative covariate (Additional file 1: Figure S12). However, since this procedure was not used in [18] and is not currently mentioned in the software documentation, we have excluded it from our primary analyses. The authors responded positively to the recommendation of documenting this procedure in future releases of the package.

#### lfdr and adapt-glm are sensitive to the number of tests

We found that the power of some methods was more sensitive to the number of hypothesis tests in the simulation studies than others. Specifically, lfdr and adapt-glm performed poorly in terms of TPR when there were fewer than 1000 tests (Fig. 3B, left panel). The lfdr result may again be due to our choice of the number of groups used in the method, as described above. We also note that the improvement in TPR of ihw over bh was not apparent without at least several thousand tests (Additional file 1: Figure S4D).

### Applicability

To investigate the applicability of modern methods to a variety of analyses and datasets, we used a combination of simulation settings and empirical case studies.

Specifically, we evaluated the performance under several different test statistic and effect size distributions in simulation. We considered normal *t* with both 5 and 11 degrees of freedom and *χ*^2^ test statistics with 4 degrees of freedom as in [16]. Additionally, we considered several different effect size distributions, ranging from unimodal to bimodal.

We also investigated the application of these methods to a series of six case studies in computational biology, including differential expression testing in bulk RNA-seq, differential expression testing in single-cell RNA-seq, differential abundance testing and correlation analysis in 16S microbiome data, differential binding testing in ChIP-seq, genome-wide association testing, and gene set analysis. These results, along with a practical discussion of the selection and assessment of informative covariates, are included in the following sections.

#### ashq and fdrreg-t are sensitive to the sampling distribution of the test statistic

**Fig. 4:**
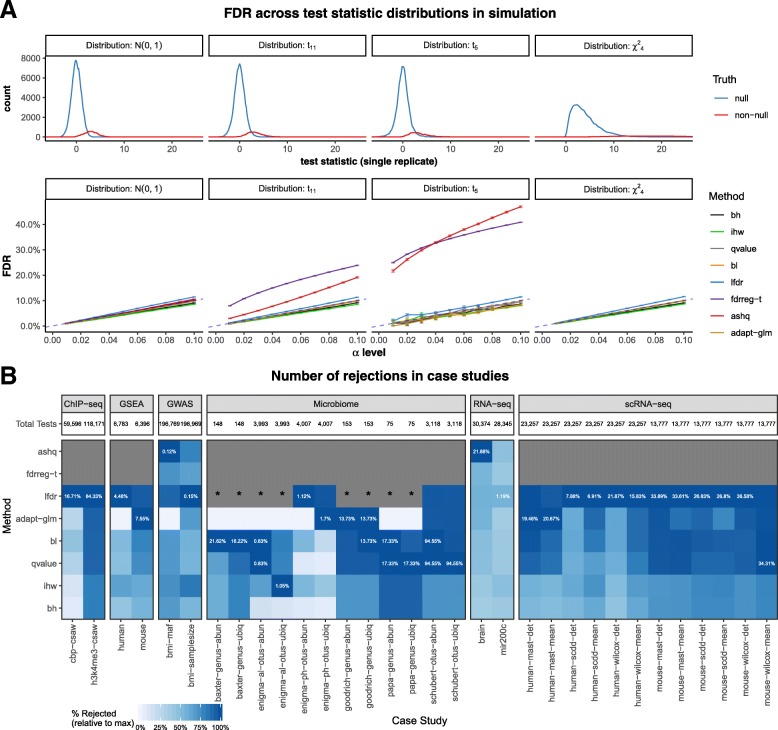
Applicability of benchmarked methods to various test statistics and case study datasets. **a** The top panel displays various null and non-null test statistic distributions used in simulations, with the corresponding observed FDR shown in the bottom panel. Note that although fdrreg-t requires normally distributed test statistics, it is included in the evaluation of *t*_11_ and *t*_5_ statistics to illustrate the effect of a heavy tailed distribution. In addition, neither ashq nor fdrreg-t is evaluated on *χ*^2^ statistics, as they violate the assumptions of the method. **b** Proportion of maximum rejections (color) for each dataset and informative covariate (column, grouped by case study) and FDR correction method (row). In each column, the maximum proportion of rejections out of the total possible number of comparisons is displayed. Where ashq and fdrreg-t could not be applied to a case study to due violation of assumptions, the cell is colored in gray. Where the method lfdr was not applied due to practical limitations on the number of tests, the cell is colored grey and marked with “*.” The informative covariate used in each case study is listed in Table 1. For case studies with more than one covariate, the covariate is denoted in the *x*-axis labels

Many of the modern FDR-controlling methods make assumptions regarding a valid distribution of *p* values. However, some methods also make assumptions about the distribution of the test statistic or effect size. Specifically, FDRreg and ASH both assume that test statistics are normally distributed [19, 20]. However, ASH is also described as being applicable to *t*-distributed statistics, although currently only based on a rough approximation [20]. The option to specify the degrees of freedom for *t*-distributed statistics based on this approximation was used for the ashq implementation in the *t*-distributed simulations. The sensitivity of these methods along with the others to changes in the underlying distributions of the test statistics was investigated through simulations across the four distributions described above. These simulation results are shown in Fig. 4a and Additional file 1: Figure S6. Since the assumptions for both FDRreg and ASH are strongly violated with *χ*^2^ test statistics, these methods were not applied in this setting.

**Table 1:** Independent and informative covariates used in case studies

Case study	Covariates found to be independent and informative
Microbiome	**Ubiquity**: the proportion of samples in which the feature is present. In microbiome data, it is common for many features to go undetected in many samples.
	**Mean nonzero abundance**: the average abundance of a feature among those samples in which it was detected. We note that this did not seem as informative as ubiquity in our case studies.
GWAS	**Minor allele frequency**: the proportion of the population which exhibits the less common allele (ranges from 0 to 0.5) represents the rarity of a particular variant.
	**Sample size (for meta-analyses)**: the number of samples for which the particular variant was measured.
Gene set analyses	**Gene set size**: the number of genes included in the particular set. Note that this is not independent under the null for over-representation tests, however (see Additional file 1: Supplementary Results).
Bulk RNA-seq	**Mean gene expression**: the average expression level (calculated from normalized read counts) for a particular gene.
Single-Cell RNA-seq	**Mean nonzero gene expression**: the average expression level (calculated from normalized read counts) for a particular gene, excluding zero counts.
	**Detection rate**: the proportion of samples in which the gene is detected. In single-cell RNA-seq it is common for many genes to go undetected in many samples.
ChIP-seq	**Mean read depth**: the average coverage (calculated from normalized read counts) for the region
	**Window Size**: the length of the region

We observed that FDR control for most methods, namely those which take *p* values as input rather than *z*-scores or effect sizes (Fig. 1), was not sensitive to the distribution of test statistics (Fig. 4a and Additional file 1: Figure S6B-C). However, violation of the normal assumption of fdrreg-t led to inflated FDR when the test statistics were *t*-distributed, and as expected, the increase in FDR was greater for the heavier-tailed *t* distribution with fewer degrees of freedom (Fig. 4a). Although it accommodates *t*-distributed test statistics, inflated FDR was also observed for ashq (both with and without specifying the correct degrees of freedom). While not included in our comparisons, the authors of [20] have recently proposed an adaptation of ashq for this case based on moderated standard error estimates using the limma R package which may provide better FDR control [32, 33].

#### ashq is not sensitive to violation of the unimodal assumption

In addition to the distributional assumptions on the test statistic, ASH assumes that the distribution of the true (unobserved) effect sizes is unimodal, referred to as the “unimodal assumption.” To investigate ASH’s sensitivity to the unimodal assumption, multiple distributions of the effect sizes were considered in both simulations and yeast in silico experiments. While most simulations included effect size distributions with most non-null effects away from zero, simulations were also performed following the unimodal assumption of ASH with a set of effect size distributions described in [20] (Additional file 1: Figures S6A, S7 and S8). In the yeast in silico experiments, two conditions were also investigated—a unimodal and a bimodal case.

We observed that even when the unimodal assumption of ASH was violated in simulation, ashq had only a slight inflation in FDR and comparable TPR to other methods (Additional file 1: Figure S7B-C). This was also observed in the yeast in silico experiments (Additional file 1: Figure S3).

#### Not all methods can be applied to every case study

We discovered that some methods could not be applied to some case studies due to restrictive assumptions. For example, FDRreg could only be applied if the tests under consideration yielded approximately normally distributed statistics. As a result, FDRreg was applied to the bulk RNA-seq and GWAS studies, but not considered in any of the other case studies since the test statistics are decidedly not normal. Likewise, ASH could only be applied if both an effect size and corresponding standard error for each test was available. As a result, ASH was excluded from case studies involving tests that only output a *p*-value or test statistic, such as permutation tests or the Wilcoxon rank-sum test (Fig. 4b). Further, the LFDR method was not applied to 3 microbiome datasets where there were fewer than 4000 total tests (200 tests per bin).

In the in silico experiments and simulations described above where the underlying properties of the data are known, it is easy to verify whether the assumptions of each method are satisfied. In practice, however, some assumptions are difficult or even impossible to check. For example, while it is feasible to assess the overall unimodality of the observed effect sizes for input to ashq, it is impossible to check the unimodal assumption for the true (unobserved) effects. For this reason, it is possible that the assumptions of ashq could be violated in some of the case studies.

#### Choice of independent covariate is application-dependent

Several covariates have been suggested for *t* tests, rank-based tests, RNA-seq DE analysis, eQTL analysis, GWAS, and quantitative proteomics [15]. In the case studies, we selected covariates based on these suggestions, as well as our own hypotheses about covariates that could potentially contain information about the power of a test, or the prior probability of a test being non-null (Table 1). We observed that the relationship between the covariates explored in the case studies and the proportion of tests rejected was highly variable (Additional file 1: Figure S13).

To select covariates for each case study, we visually evaluated whether each covariate was informative by examining a scatter plot of the independent covariate percentile and the *p*-value. If this contained any noticeable trend such that certain values of the informative covariate were enriched for smaller *p* values, we considered the covariate to be informative.

We also visually evaluated whether each covariate was approximately independent under the null hypothesis following the recommendations of [15]. Specifically, we examined the histogram of *p* values stratified by small, moderate, and large values of the covariate (Additional file 1: Figure S14). If the distribution of the moderate to large *p* values appeared approximately uniform, we considered the covariate to be approximately independent of the *p* values under the null hypothesis.

For almost all choices of the covariate originally considered, we were able to substantiate evidence for informativeness and independence. One notable exception was the set size covariate for the overrepresentation test in the gene set analysis case study. Here, we found that although the covariate appeared to be informative, it was not independent under the null hypothesis (Additional files 22 and 23). We observed a dependence in the global enrichment of smaller *p* values in small gene sets. This is a direct consequence of the fact that a single DE gene represents a larger proportion of a smaller gene set than it does a larger gene set. As a result, we only show the results for gene set analysis using gene set enrichment analysis (GSEA), which, unlike the overrepresentation test, does not rely on selecting a subset of DE genes. Instead, GSEA incorporates the rank of every gene into the evaluation of a gene set. The gene set size covariate did satisfy the independent and informative criteria for *p* values obtained from GSEA.

### Consistency

We observed that the relative performance of modern methods differed depending on the particular scenario. To evaluate the consistency of the performance of modern methods, we summarized the variability across the different simulation studies, in silico experiments, and case studies.

**Fig. 5:**
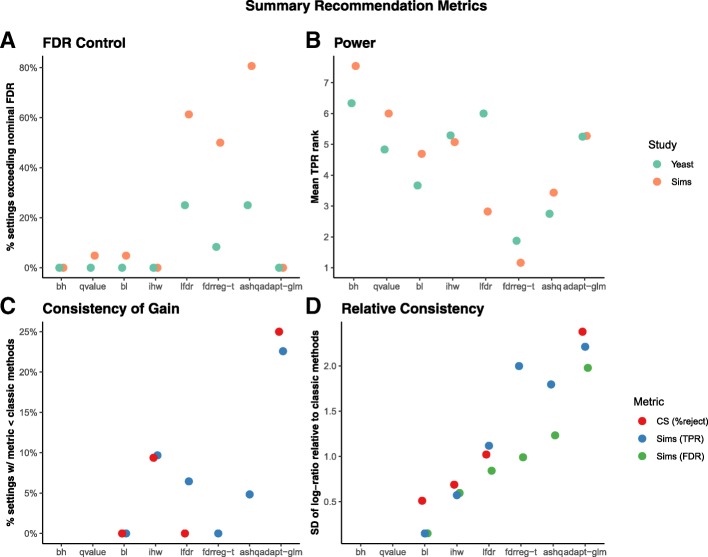
Summary metrics computed to rate methods for final recommendations. Several metrics were computed over all settings of the simulations, and yeast experiments, as well as all datasets and covariates in the case studies to evaluate the **a** FDR control, **b** power, and **c**, **d** consistency of the evaluated methods. In **a** and **b**, color denotes whether the metric is computed over simulations (Sims) or yeast in silico experiments (Yeast). In **c** and **d**, color denotes whether the metric is computed for TPR or FDR in the simulations (“Sims (TPR)” and “Sims (FDR)”, respectively), or for the percentage of rejections in the case studies “CS (%reject).” In all panels, the methods are shown on the *x*-axis, and the methods with superior performance are those with a low value of the *y*-axis metric. Cutoffs used with the metrics shown are provided in the “Methods” section

Across all simulation studies and yeast in silico experiments, we quantified the overall proportion of settings of modern FDR methods achieving FDR control (Fig. 5a) and the average ranking of TPR (Fig. 5b). In addition, we quantified the variability across simulation settings of modern FDR methods relative to classic methods (Fig. 5c, d). We also evaluated the consistency of the number of rejections in case studies both with and without informative covariates. Note that variability across case studies was not evaluated for fdrreg and ashq, as the methods were only applied to a subset of the datasets. Detailed discussion of these results is provided in the following sections.

#### Consistency of FDR and power

We observed that adapt-glm, ihw, and bl achieved FDR control in almost all simulation and in silico experiment settings (Fig. 5a) and were on average ranked near the median of all methods in terms of TPR (Fig. 5b). However, adapt-glm frequently resulted in lower TPR than classic methods (Fig. 5c) and had the highest variability of TPR and FDR across all simulation settings (Fig. 5d). Note that although ihw had lower TPR than bh and qvalue in about 10% of simulation settings (Fig. 5c), this difference was usually small and the variability of ihw relative to classic methods was smaller than most other modern methods (Fig. 5d).

On the other hand, fdrreg-t and ashq were consistently ranked among the top methods in terms of TPR (Fig. 5b), but both failed to control FDR in more than 40% of simulation settings (Fig. 5b) and exhibited higher variability of both FDR and TPR than bl and ihw (Fig. 5d). lfdr showed similar performance to ashq and fdrreg-t but was ranked more favorably in terms of TPR in simulation studies compared to in silico experiments (Fig. 5).

#### Number of rejections are highly variable in case studies

In the case studies, we found that lfdr and ashq (where applied) made the most rejections on average (Additional file 1: Figure S15), a similar trend to that observed in the yeast in silico simulations (Additional file 1: Figure S16). Otherwise, the relative ranking among the methods varied among datasets and covariates used in each analysis (Fig. 4b).

The adapt-glm and lfdr methods had the most variable performance relative to classic methods across case studies (Fig. 5d). In particular, adapt-glm rejected fewer tests than the classic methods in approximately 25% of case study datasets (Fig. 5c). The performance pattern of bl was very similar to qvalue (Fig. 4). Likewise, ihw exhibited similar patterns to bh. The ashq method, where applied, was usually among the methods with the most rejections, and bh consistently found among the fewest discoveries on average among all FDR-controlling methods.

#### Gain over uninformative covariates is highly variable in case studies

To investigate how each method uses information from covariates and to assess performance in the case that a covariate is completely uninformative, we also included a randomly generated uninformative covariate in each case study that was independent of the *p* values under the null and alternative.

The average gain from using an informative covariate as compared to an uninformative covariate was usually modest but, in rare cases, resulted in order of magnitude differences (Additional file 1: Figure S11A). The gain was also highly variable across case studies, covariates, and datasets. In some cases, the adapt-glm and bl methods made fewer rejections using the informative covariate (Additional file 1: Figure S17).

## Discussion and conclusions

**Fig. 6:**
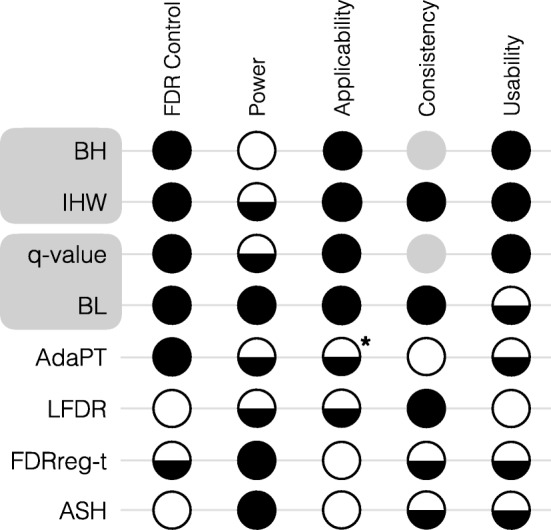
Summary of recommendations. For each method (row) and evaluation criteria (column), a filled circle denotes the method was superior, a half-filled circle denotes the method was satisfactory, and an empty circle denotes the method was unsatisfactory. Gray circles are used to denote that BH and qvalue were not evaluated for the consistency criteria. An asterisk is used to denote that applicability was assessed slightly differently for AdaPT. Detailed evaluation criteria are provided in the “Methods” section

We have presented a systematic evaluation to guide researchers in their decisions regarding the methods to control for false discoveries in their own data analysis. A series of case studies and simulations were performed to investigate which methods maximize the number of discoveries while controlling FDR at the nominal *α*-level. We conclude by highlighting several key results and practical recommendations, which are summarized in Fig. 6.

We found that modern methods for FDR control were more powerful than classic approaches, but the gain in power was generally modest. In addition, with the exception of AdaPT, most methods that incorporate an independent covariate were not found to underperform classic approaches, even when the covariate was completely uninformative. Because adapt-glm sometimes performed worse with the use of a covariate, we recommend including a null model as an input along with the covariate model when applying AdaPT.

Overall, we found the performance of the modern FDR methods generally improved over the classic methods as (1) the informativeness of the covariate increased, (2) the number of hypothesis tests increased, and (3) the proportion of non-null hypotheses increased. Although it is not possible to assess (1) and (3) in practice, most methods still controlled FDR when the covariate was weakly informative and the proportion of non-nulls was high.

Across our simulation and case study evaluations, we found that IHW and BL generally had the most consistent gains in TPR over classic methods, while still controlling the FDR (Fig. 6). While the TPR of BL was often higher than IHW, we note that the gain in power of BL relative to IHW should be interpreted in light of any gain in power of qvalue to BH, due to the special relationship between these pairs of methods. Specifically, IHW and BL reduce to BH and *q*value, respectively, when the covariate is uninformative. The power of IHW was generally superior to BH when the covariate was sufficiently informative, but almost identical to BH when the covariate was not informative enough or when there were only a few thousand tests. Likewise, the power of BL was generally superior to Storey’s *q*-value when the covariate was sufficiently informative and had a monotonic relationship with the probability of a test being non-null.

We also found that although the majority of methods performed similarly in controlling the FDR, some methods were not able to control FDR at the desired level under certain settings. This occurred for empirical FDRreg when the proportion of non-nulls was near 50%, LFDR when there were fewer than 5000 tests, and ASH when the test statistic was *t*-distributed.

We have provided several useful examples of how to use an informative covariate in biological case studies. When choosing a covariate for a particular analysis, it is important to evaluate whether it is both informative and independent under the null hypothesis. In other words, while the covariate should be informative of whether a test is truly positive, if a test is truly negative, knowledge of the covariate should not alter the validity of the *p-*value or test statistic. Violation of this condition can lead to loss of type I error control and an inflated rate of false discoveries [34]. To avoid these pitfalls, we recommend using previously proposed visual diagnostics to check both the informativeness and independence of the selected covariate [15].

We also note that although in this study we only considered a single (univariate) covariate in the simulations and case studies, some of the modern methods are able to incorporate multiple covariates. BL, AdaPT, and FDRreg can all accommodate an arbitrary set of covariates through the specification of a design matrix. In particular, AdaPT is well-suited to high-dimensional problems, as it provides an implementation that uses *L*_1_-penalized generalized linear models for feature selection. Further investigation is needed in the selection of multiple covariates and the potential gain in performance over using a single covariate.

Finally, we rank IHW, BH, and Storey’s *q*-value as superior in terms of user-friendliness and documentation, critical for lasting use and impact in the community. All methods were implemented and evaluated in R. With the exception of LFDR and the latest version of FDRreg, methods were easily accessible from packages in CRAN or Bioconductor, the primary repositories for R packages. Implementing LFDR and installing FDRreg both required additional work (see the “Methods” section). In their implementations, most methods provide direct measures, such as adjusted *p* values or *q* values, as outputs directly to users. In contrast, bl provides null hypothesis weights which must be manually applied to BH-adjusted *p* values by the user to control FDR. In addition, bl, adapt-glm, and fdrreg all require specifying a functional relationship between the covariate and null proportion in the form of a model or formula. While this provides the user significant flexibility, it can also be unintuitive for researchers not familiar with the underlying modeling frameworks of these methods. A benchmarking experiment to determine reasonable default values for parameters to improve the user-friendliness of these methods is left as future work.

## Methods

### Assessing assumptions

ASH and FDRreg differ substantially from the remaining methods, and care should be taken to verify that the appropriate inputs are available and that the underlying assumptions are indeed valid. Based on these criteria, both methods were excluded from most case studies and several simulation settings considered in this benchmark. A more detailed discussion of these assumptions is included below. For FDRreg and adapt-glm, the informative covariate must be specified in the form of a model matrix or formula (respectively). In both cases, we use the same type of formula or model matrix used in the authors’ original publications [18, 19]. For lfdr, the informative covariate must be a discrete group label. We follow the implementation of lfdr developed in [15], which automatically bins the input covariate into 20 approximately equally sized bins before estimating the within-group local FDR (source code to implement this procedure available on the GitHub repository linked in “Availability of data and materials” section [35]).

Common to all modern FDR-controlling procedures included in Fig. 1 is the requirement that the informative covariate also be independent of the *p*-value or test statistic under the null. That is, while the covariate should be informative of whether a test is truly positive, if a test is truly negative, knowledge of the covariate should not alter the validity of the *p*-value or test statistic. Violation of this condition can lead to loss of type I error control and an inflated rate of false discoveries [34]. To avoid these pitfalls, previously proposed visual diagnostics were used to check both the informativeness and independence of the selected covariate [15].

### Implementation of benchmarked methods

All analyses were implemented using R version 3.5.0 [24]. We used version 0.99.2 of the R package SummarizedBenchmark [36] to carry out the benchmark comparisons, which is available on GitHub at the “fdrbenchmark” branch at https://github.com/areyesq89/SummarizedBenchmark/tree/fdrbenchmark[37].

While other modern FDR-controlling methods have also been proposed, methods were excluded if accompanying software was unavailable or if the available software could not be run without substantial work from the user [38].

#### BH

Adjusted *p* values by BH were obtained using the p.adjust function from the stats base R package, with option method=~BH~.

#### q value

Storey’s *q*-values were obtained using the qvalue function in version 2.12.0 of the qvalue Bioconductor R package.

#### IHW

Adjusted *p* values by IHW were obtained using the adj _pvalues function on the output of the ihw function, both from version 1.8.0 of the IHW Bioconductor R package.

#### BL

Adjusted *p* values by BL were obtained by multiplying BH adjusted *p* values (see above) by the *π*_0,*i*_ estimates obtained using the lm _pi0 function from version 1.6.0 of the swfdr Bioconductor R package.

#### lfdr

Adjusted *p* values by lfdr were obtained by first binning the independent covariate into 20 approximately equal-sized groups using the groups _*by*_filter function the IHW R package. Next, the fdrtool function from version 1.2.15 of the fdrtool CRAN R package was applied to the *p* values within each covariate bin separately, with the parameter statistic="pvalue". We require that at least 200 tests per bin, as recommended by fdrtool. Note that we follow [15] and use fdrtool rather than the locfdr package recommended by [17] to obtain local false discovery rates, as the former may operate directly on *p* values instead of requiring *z*-scores as in the latter.

#### FDRreg

For applications where the test statistic was assumed to be normally distributed, Bayesian FDRs were obtained by the FDRreg function from version 0.2-1 of the FDRreg R package (obtained from GitHub at https://github.com/jgscott/FDRreg). The features parameter was specified as a model matrix with a B-spline polynomial spline basis of the independent covariate with 3 degrees of freedom (using the bs function from the splines base R package) and no intercept. The nulltype was set to "empirical" or "theoretical" for the empirical and theoretical null implementations of FDRreg, respectively.

#### ASH

*q* values were obtained using the get _qvalue function on the output of the ash function, both from version 2.2-7 of the ashr R CRAN package. The effect sizes and their corresponding standard errors were input as the effect _size and sebetahat parameters, respectively.

#### AdaPT

*q* values were obtained using the adapt _glm function version 1.0.0 of the adaptMT CRAN R package. The pi _formulas and mu _formulas arguments were both specified as natural cubic B-spline basis matrices of the independent covariate with degrees of freedom ∈{2,4,6,8,10} (using the ns function from the splines base R package).

### Yeast in silico experiments

Preprocessed RNA-seq count tables from [29] were downloaded from the authors’ GitHub repository at https://github.com/bartongroup/profDGE48. All samples that passed quality control in the original study were included. All genes with a mean count of at least 1 across all samples were included, for a total of 6553 genes. Null comparisons were constructed by randomly sampling 2 groups of 5 and 10 samples from the same condition (Snf2-knockout). Non-null comparisons of the same size were constructed by adding differentially expressed (DE) genes in silico to null comparisons. In addition to different sample sizes, several different settings of the proportion of non-null genes, the distribution of the non-null effect sizes and informativeness of the covariate were explored. An overview of the different settings is provided in Additional file 1 :Table S2.

We evaluated the results using a low proportion of non-null genes (500, or approximately 7.5% non-null) as well as a high proportion (2000 or approximately 30% non-null). The non-null genes were selected using probability weights sampled from a logistic function (where weights $w(u)=\frac {1}{1+e^{-10u+5}}$, and *u*∼*U*(0,1)). Three types of informative covariates were explored: (1) strongly informative, (2) weakly informative, and (3) uninformative. The strongly informative covariate *X*_*s*_ was equal to the logistic sampling weight *w*. The weakly informative covariate *X*_*w*_ was equal to the logisitic sampling weight plus noise: *w*+*ε*, where *ε*∼*N*(0,0.25), truncated such that *X*_*w*_∈[0,1]. The uninformative *X*_*u*_ covariate was unrelated to the sampling weights and drawn from a uniform distribution such that *X*_*u*_∼*U*(0,1).

We also evaluated the results under two different distributions of non-null effect sizes: (1) unimodal and (2) imodal. For unimodal alternative effect size distributions, the observed fold changes for the selected non-null genes in a non-null empirical comparison of the same sample size were used. For bimodal alternatives, observed test statistics *z* from an empirical non-null comparison of the same sample size were sampled with probability weights *w*(*z*)=*f*(|*x*|;*α*,*β*), where *f* is the Gamma probability density function (with shape and rate parameters *α*=4.5 and *β*=1−1*e*^−4^, respectively). The corresponding effect sizes (fold changes, *FC*) for ashq were calculated assuming a fixed standard error: *FC*=*zσ*_*m*_, where *σ*_*m*_ is the median standard error of the *log*_2_ fold change across all genes.

To add differential signal to the designated non-null genes, the expression in one randomly selected group was then multiplied by their corresponding fold change. Differential expression analysis using DESeq2 [39] was carried out on both the null and non-null comparisons to assess specificity and sensitivity of the FDR correction methods. Genes for which DESeq2 returned NA*p* values were removed. In each setting, simulations were repeated 100 times and the average and standard error are reported across replications. The results displayed in the main manuscript contain 2000 DE genes, use the strongly informative covariate, and have a sample size of 5 in each group. The results for all settings are presented in Additional files 2, 3, 4, and 5.

### Polyester in silico experiments

The yeast RNA-seq data described in the previous section was used to estimate the model parameters using version 1.16.0 of the polyester [28] R Bioconductor package. All samples that passed quality control in the original study were included. A baseline group containing all the samples in the wild-type group was used, and genes with mean expression of less than 1 count were filtered out. Counts were library size normalized using DESeq2 size factors [39], and the get_params function from the polyester package was used to obtain the model parameters. Counts were simulated using the create_read_numbers function. Using the same sample sizes as the yeast in silico experiments (5 or 10 samples in each group), we evaluated a null comparison, where the beta parameters of the create_read_numbers function (which represent effect size) were set to zero for all genes. We also evaluated non-null comparisons where the beta parameters were drawn from a standard normal distribution for 2000 non-null genes. The non-null genes were selected in the same way as the yeast in silico experiments described in the previous section. Differential expression analysis and evaluation of FDR correction methods was also carried out as described for the yeast experiments. Results are presented in Additional file 6.

### Simulation studies

We performed Monte Carlo simulation studies to assess the performance of the methods with known ground truth information. In each simulation, *M* observed effect sizes, $\{d_{i}\}_{i=1}^{M}$, and standard errors, $\{s_{i}\}_{i=1}^{M}$, were sampled to obtain test statistics, $\{t_{i} = d_{i} / s_{i}\}_{i=1}^{M}$. Letting $\{\delta _{i}\}_{i=1}^{M}$ denote the true effect sizes, each *t*_*i*_ was tested against the null hypothesis $H_{0}^{i}: \delta _{i} = 0$. Observed effect sizes and standard errors were simulated to obtain test statistics following one of four distributions under the null:

- Standard normal distribution
- *t* distribution with 11 degrees of freedom
- *t* distribution with 5 degrees of freedom
- *χ*^2^ distribution with 4 degrees of freedom.

For each test *i*, let *h*_*i*_ denote the true status of the test, with *h*_*i*_=0 and *h*_*i*_=1 corresponding to the test being null and non-null in the simulation. True effect sizes, *δ*_*i*_, were set to 0 for {*i*|*h*_*i*_=0} and sampled from an underlying non-null effect size distribution for {*i*|*h*_*i*_=1}. For normal and *t* distributed test statistics, observed effect sizes were simulated by adding standard normal noise to the true effect sizes such that *d*_*i*_∼*N*(*δ*_*i*_,1). The standard errors were all set to 1 to obtain normal test statistics and set to $s_{i} = \sqrt {v_{i} / \nu }$ with each $v_{i}\sim \chi ^{2}_{\nu }$ independent to obtain *t* statistics with *ν* degrees of freedom. For *χ*^2^ test statistics, observed effect sizes were sampled from non-central *χ*^2^ distributions with non-centrality parameters equal to the true effect sizes such that $d_{i} \sim \chi ^{2}_{4}(ncp = \delta _{i})$. Standard errors were not used to simulate *χ*^2^ statistics and were simply set to 1. The *p*-value was calculated as the two-tail probability of the sampling distribution under the null for normal and *t* statistics. The upper-tail probability under the null was used for *χ*^2^ statistics.

In all simulations, independent covariates, $\{x_{i}\}_{i = 1}^{M}$, were simulated from the standard uniform distribution over the unit interval. In the uninformative simulation setting, the $\{h_{i}\}_{i=1}^{M}$ were sampled from a Bernoulli distribution according to the marginal null proportion, $\bar \pi _{0}$, independent of the {*x*_*i*_}. In all other settings, the $\{h_{i}\}_{i=1}^{M}$ were sampled from Bernoulli distributions with test-specific probabilities determined by the informative covariates through a function, *p*(*x*_*i*_), taking values in [0,1]. Several forms of *p*(*x*_*i*_) were considered in the simulations. The *p*(*x*_*i*_) were chosen to explore a range of relationships between the covariate and the null probability of a test. For further flexibility, the functional relationships were defined conditional on the marginal null probability, $\bar \pi _{0}$, so that similar relationships could be studied across a range of $\bar \pi _{0}$. The following $p(x_{i};\bar \pi _{0})$ relationships, shown in Additional file 1: Figure S5A for $\bar \pi _{0} = 0.90$, were investigated in the simulations.

$$\begin{array}{*{20}l} p^{\text{cubic}}(x;\bar\pi_{0}) &= 4(1-\pi_{0})(1-x)^{1/3} + 4 \pi_{0} - 3 \\ p^{\text{step}}(x;\bar\pi_{0}) &= \left\{ \begin{array}{ll} \bar\pi_{0}/2 - 1/2 & \text{if } x \in [0, 1/4) \\ \bar\pi_{0}/4 - 1/4 & \text{if } x \in [1/4, 1/2) \\ -\bar\pi_{0}/4 + 1/4 & \text{if } x \in [1/2, 3/4) \\ -\bar\pi_{0}/2 + 1/2 & \text{if } x \in [3/4, 1] \end{array}\right. \\ p^{\text{sine}}(x;\bar\pi_{0}) &= \left\{ \begin{array}{l} \bar\pi_{0} - \bar\pi_{0} \sin(2\pi \cdot x) \\ \ \ \ \ \ \ \ \ \ \ \ \ \ \ \ \ \ \text{if } \bar\pi_{0} \in [0, 1/2) \\ \bar\pi_{0} + (1-\bar\pi_{0}) \sin(2\pi \cdot x) \\ \ \ \ \ \ \ \ \ \ \ \ \ \ \ \ \ \ \text{if } \bar\pi_{0} \in [1/2, 1] \end{array}\right. \\ p^{\text{cosine}}(x;\bar\pi_{0}) &= \left\{ \begin{array}{l} \bar\pi_{0} - \bar\pi_{0} \cos(2\pi \cdot x) \\ \ \ \ \ \ \ \ \ \ \ \ \ \ \ \ \ \ \text{if } \bar\pi_{0} \in [0, 1/2) \\ \bar\pi_{0} + (1-\bar\pi_{0}) \cos(2\pi \cdot x) \\ \ \ \ \ \ \ \ \ \ \ \ \ \ \ \ \ \ \text{if } \bar\pi_{0} \in [1/2, 1] \end{array}\right. \end{array} $$

The functions *p*^cubic^ and *p*^step^ are valid, i.e., map to [0,1], for $\bar \pi _{0}\in [3/4, 1]$ and $\bar \pi _{0}\in [1/2,1]$, respectively. All other *p*(*x*_*i*_) are valid for $\bar \pi _{0}\in [0,1]$. In addition to these functional relationships, we also considered two specialized relationships with $\bar \pi _{0}$ fixed at 0.80. These specialized relationships were parameterized by an informativeness parameter, *δ*∈[0,1], such that when *δ*=0, the covariate was completely uninformative and stratified the hypotheses more effectively as *δ* increased.

$$\begin{array}{*{20}l} p^{\text{c-info}}(x;\delta) &= 0.8 \cdot (1 - \delta) + \delta / (1+e^{5 - 25x}) \\ p^{\text{d-info}}(x;\delta) &= \left\{ \begin{array}{ll} 0.2 \cdot (4 + \delta) & \text{if } x \in [0, 4/5] \\ 0.8 \cdot (1 - \delta) & \text{if } x \in (4/5, 1] \end{array}\right. \end{array} $$

The first, *p*^c-info^, is a continuous relationship between the covariate, *x* and the null proportion, *π*_0_, while the second, *p*^d-info^, is discrete. The results of simulations with *p*^c-info^ are shown in Additional file 1: Figure S4A-B, where the “informativeness” axis is simply 100·*δ*. The covariate relationships, *p*^c-info^ and *p*^d-info^, are shown in Additional file 1: Figure S9 across a range of *δ* informativeness values.

Simulations were formulated and performed as several distinct case studies, with full results presented in Additional files 7, 8, 9, 10, 11, 12, 13, 14, 15, 16, 17, 18, 19, and 20. The complete combination of settings used in each simulation case study is given in Additional file 1: Table S3. In each case study, simulations were repeated 100 times, and performance metrics are reported as the average across the replications.

FDRreg and ASH were excluded from simulations with *χ*^2^ distributed test statistics because the setting clearly violated the assumptions of both methods (Fig. 1).

### Case studies

We explored several real case studies using publicly available datasets. Unless otherwise stated, we use an *α* = 0.05 level to define a positive test. In all cases, we also evaluate the results using an independent but uninformative covariate (sampled from a standard uniform distribution). The locations of where the data can be found and the main details for each case-study is described below. For more specific analysis details, please refer to Additional files 21, 22, 23, 24, 25, 26, 27, 28, 29, 30, 31, 32, 33, 34, 35, 36, 37, 38, 39, 40, and 41 which contain complete reproducible code sets.

#### Genome-wide association study

GWAS analysis results were downloaded from http://portals.broadinstitute.org/collaboration/giant/images/3/3a/BMI.SNPadjSMK.zip[40, 41]. We used the results subset by European ancestry provided in the file BMI.SNPadjSMK.CombinedSexes.EuropeanOnly.txt to avoid the impact of population stratification on our results. We followed [42] and implemented a linkage disequilibrium (LD)-based pruning step (using the clump command of PLINK v1.90b3s [43]) to remove SNPs in high LD (*r*^2^<0.2) with any nearby SNP (< 250 Kb), based LD estimates from the 1000 Genomes phase three CEU population data [44], available at http://neurogenetics.qimrberghofer.edu.au/iSECA/1000G_20101123_v3_GIANT_chr1_23_minimacnamesifnotRS_CEU_MAF0.01.zip. We explored the use of both sample size and minor allele frequency for each SNP as informative covariates. For fdrreg-e and fdrreg-t, which require a normally distributed test statistic as input, the *t* statistic (effect size divided by standard error) was used. Because of the large sample sizes in this study (median 161,165), the *t* statistics were approximately normal. For ashq, we used the provided effect size and standard error of the test statistics. Full results are provided in Additional file 21.

#### Gene set analysis

We used two RNA-seq datasets that investigated the changes in gene expression (1) between the cerebellum and cerebral cortex of 5 males from Genotype-Tissue Expression Project [45] and (2) upon differentiation of hematopoietic stem cells (HSCs) into multipotent progenitors (MPP) [46] (processed data for both available at 10.5281/zenodo.1475409 [47]). For the independent and informative covariate, we considered the size of the gene sets. We considered two different gene set analysis methods: gene set enrichment analysis (GSEA) [48] and overrepresentation testing [49]. To implement the overrepresentation test, we first used version 1.20.0 of the DESeq2 R Bioconductor package to obtain a subset of differentially expressed genes (with adjusted *p*-value < 0.05), on which a test of overrepresentation of DE genes among gene sets was performed using version 1.32.0 of the goseq R Bioconductor package [49]. To implement GSEA, we used version 1.6.0 of the fgsea R Bioconductor package [50] and used the DESeq2 test statistics to rank the genes. For both methods, Gene Ontology categories obtained using version 2.36.1 of the biomaRt R Bioconductor package containing at least 5 genes were used for the gene sets. For fgsea, 10,000 permutations were used and gene sets larger than 500 genes were excluded as recommended in the package documentation. The methods fdrreg-e, fdrreg-t, and ashq were excluded since they require test statistics and/or standard errors that GSEA does not provide. For goSeq, we also filtered on gene sets containing at least one DE gene. Full results are provided in Additional files 22, 23, 24, and 25.

#### Bulk RNA-seq

We used two RNA-seq datasets to asses the performance of modern FDR methods in the context of differential expression. The first dataset consisted of 20 paired samples of the GTEx project. These 20 samples belonged to 2 tissues (Nucleus accumbens and Putamen) of 10 female individuals. These samples were preprocessed as described in [51] (processed data available at 10.5281/zenodo.1475409 [47]). We used a second dataset from an experiment in which the microRNA *mir200c* was knocked down in mouse cells [52]. The transcriptomes of knockdown cells and control cells were sequenced in biological duplicates. The processed samples of the knockdown experiment were downloaded from the *recount2* database available at http://duffel.rail.bio/recount/v2/SRP030475/rse_gene.Rdata [53, 54]. For each dataset, we tested for differential expression using DESeq2. For FDR methods that can use an informative covariate, we used mean expression across samples, as indicated in the DESeq2 vignette. Full results are provided in Additional files 26 and 27.

#### Single-cell RNA-seq

We selected two datasets from the *conquer* [55] database available at http://imlspenticton.uzh.ch/robinson_lab/conquer/data-mae/GSE84465.rdsand http://imlspenticton.uzh.ch/robinson_lab/conquer/data-mae/GSE94383.rds [56]. First, we detected differentially expressed genes between glioblastoma cells sampled from a tumor core with those from nearby tissue (GSE84465) [57]. In addition, we detected DE genes between murine macrophage cells stimulated to produce an immune response with an unstimulated population (GSE94383) [58]. We filtered out cells with a mapping rate less than 20% or fewer than 5% of genes detected. Genes detected in at least 5% of cells were used in the analysis and spike-in genes were excluded. We carried out DE analyses using two different methods developed for scRNA-seq: scDD [59] and MAST [60], along with the Wilcoxon rank-sum test. MAST was applied to log2(TPM + 1) counts using version 1.6.1 of the MAST R Bioconductor package. scDD was applied to raw counts normalized by version 1.8.2 of the scran R Bioconductor package [61] using version 1.4.0 of the scDD R Bioconductor package. Wilcoxon was applied to counts normalized by TMM (using version 3.22.3 of the edgeR R Bioconductor package [62]) using the wilcox.test function of the stats base R package. We examined the mean non-zero expression and detection rate (defined as the proportion of cells expressing a given gene) as potentially informative covariates. The fdrreg-e and fdrreg-t methods were excluded since none of the test statistics used are normally distributed. Likewise, ashq was excluded since none of the methods considered provide effect sizes and standard errors. Full results are provided in Additional files 28, 29, 30, 31, 32, and 33.

#### ChIP-seq

ChIP-seq analyses were carried out on two separate datasets. First, H3K4me3 data from two cell lines (GM12878 and K562) were downloaded from the ENCODE data portal [63] at http://hgdownload.cse.ucsc.edu/goldenpath/hg19/encodeDCC/wgEncodeBroadHistone/ [64]. In each cell line, four replicates were selected with half of them from one laboratory and the other half from another laboratory. We performed two types of differential binding analyses between cell lines. First, following the workflow of [65], we used the csaw [66] method (version 1.14.1 of the csaw Bioconductor R package) to identify candidate de novo differential windows and applied the edgeR [62] method (version 3.22.3 of the edgeR R Bioconductor package) to test for significance. Second, we tested for differential binding only in predefined promoter regions using DESeq2 [39]. Promoter regions were obtained from the UCSC “Known Gene” annotations for human genome assembly hg19 (GRCh37). The csaw-based analysis was also carried out on a second ChIP-seq dataset comparing CREB-binding protein in wild-type and CREB knockout mice [65, 67] (GSE54453) obtained from the European Nucleotide Archive at https://www.ebi.ac.uk/ena/data/view/PRJNA236594[68]. For the csaw-based analyses, we used the region width as the informative covariate. For the promoter region-based analysis, we used mean coverage as the informative covariate. The fdrreg-e and fdrreg-t methods were excluded from csaw analyses since the test statistics used are non normally distributed. Likewise, ashq was excluded since csaw does not provide effect sizes and standard errors. Full results are provided in Additional files 34, 35, and 36.

#### Microbiome

We performed two types of analyses (1) differential abundance analysis, and (2) correlation analysis. For the differential abundance analyses, we used four different datasets from the MicrobiomeHD database [69] available at 10.5281/zenodo.840333[70]: (1) obesity [71], (2) inflammatory bowel disease (IBD) [72], (3) infectious diarrhea (clostridium difficile (CDI) and non-CDI) [73], and (4) colorectal cancer (CRC) [74]. These studies were processed as described in [69]. We performed Wilcoxon rank-sum differential abundance tests on the operational taxonomic units (OTUs, sequences clustered at 100% genetic similarity) and on taxa collapsed to the genus level as in [69]. Full results are provided in Additional files 37, 38, 39, and 40.

For the correlation analyses, we used a previously published dataset of microbial samples from monitoring wells in a site contaminated by former waste disposal ponds, where all sampled wells have various geochemical and physical measurements [75]. Paired-end reads were merged using PEAR (version 0.9.10) and demultiplexed with QIIME v 1.9.1 split_libraries_fastq.py (maximum barcode error of 0 and quality score cutoff of 20) [76, 77]. Reads were dereplicated using USEARCH v 9.2.64 -fastx_uniques, and operational taxonomic units (OTUs) were called with -cluster_otus and an identity threshold of 0.97 [78]. These data were processed with the amplicon sequence analysis pipeline http://zhoulab5.rccc.ou.edu/pipelines/ASAP_web/pipeline_asap.php and are available at 10.5281/zenodo.1455793 [79]. We carried out a Spearman correlation test (*H*_0_:*ρ*=0) betwee n OTU relative abundances across wells and the respective values of three geochemical variables: pH, Al, and SO_4_. Full results are provided in Additional file 41.

For all analyses, we examined the ubiquity (defined as the percent of samples with non-zero abundance of each taxa) and mean non-zero abundance of taxa as potentially informative covariates. The results in the main manuscript are shown for the OTU level, unless there were no rejections in the vast majority of methods, and then the results are shown for genus level. The SO_4_ dataset was also excluded from the main results since most methods find no rejections. We excluded fdrreg-e, fdrreg-t, and ashq since neither the Wilcoxon nor Spearman test statistics are normally distributed, nor do they provide an effect size and standard error. Due to small numbers of tests, lfdr was excluded from the obesity and IBD genus level analyses.

### Evaluation metrics

All studies (in silico experiments, simulations, and case studies) were evaluated on the number of rejections at varying *α* levels, ranging from 0.01 to 0.10. The overlap among rejection sets for each method was examined using version 1.3.3 of the UpSetR R CRAN package. In silico experiments and simulation studies were also evaluated on the following metrics at varying *α* levels, ranging from 0.01 to 0.10: TPR, observed FDR, and true negative rate (TNR). Here, we define TPR as the number of true positives out of the total number of non-null tests, observed FDR as the number of false discoveries out of the total number of discoveries (defined as 0 when there are no discoveries), and TNR as the number of true negatives out of the total number of null tests.

### Summary metrics

The final ratings presented in Fig. 6 were determined from the aggregated results of the simulations, yeast experiments, and case studies.

#### FDR control

Ratings were determined using the results from non-null simulations where all methods were applied, i.e., excluding *χ*^2^ settings, and all non-null yeast experiments. In each setting or experiment, a method was determined to control FDR at the nominal 5% cutoff if the mean FDR across replications was less than one standard error above 5%. The following cutoffs were used to determine superior, satisfactory, and unsatisfactory methods.

- Superior: failed to control FDR in less than 10% of settings in both simulations and yeast experiments.
- Satisfactory: failed to control FDR in less than 10% of settings in either simulations or yeast experiments.
- Unsatisfactory: otherwise.

The computed proportion of simulation and yeast settings exceeding the nominal FDR threshold are shown in Fig. 5a.

#### Power

Similar to the above, ratings were determined using the results from non-null simulations where all methods were applied and all non-null yeast experiments. In each setting or experiment, methods were ranked in descending order according to the mean TPR across replications at the nominal 5% FDR cutoff. Ties were set to the intermediate value, e.g., 1.5, if two methods tied for the highest TPR. The mean rank of each method was computed across simulation settings and yeast experiments separately and used to determine superior, satisfactory, and unsatisfactory methods according to the following cutoffs.

- Superior: mean TPR rank less than 5 (of 8) in both simulations and yeast experiments.
- Satisfactory: mean TPR rank less than 6 (of 8) in both simulations and yeast experiments.
- Unsatisfactory: otherwise.

Mean TPR ranks for methods across simulation and yeast settings are shown in Fig. 5b.

#### Consistency

Ratings were determined using results from non-null simulations where all methods were applied and all case studies. Here, ashq and fdrreg-t were excluded from metrics computed using the case studies, as the two methods were only applied in 4 of the 26 case studies. In each simulation setting, the TPR and FDR of each covariate-aware method were compared against the TPR and FDR of both the BH approach and Storey’s *q*-value at the nominal 5% FDR cutoff. Similarly, in each case study, the number of rejections of each method was compared against the number of rejections of BH and Storey’s *q*-value. Based on these comparisons, two metrics were computed for each method.

First, in each setting and case study, a modern method was determined to underperform the classical approaches if the TPR or number of rejections was less than 95% of the minimum of the BH approach and Storey’s *q*-value. The proportion of simulation settings and case studies where a modern method underperformed was used to determine the consistent stability of an approach (Fig. 5c). The FDR of the methods was not used for this metric.

Second, in each simulation setting and case study, the log-ratio FDR, TPR, and number of rejections were computed against a baseline for each modern method. For each setting, the average across the classical methods was used as the baseline. Methods were then ranked according to the standard deviation of these log ratios, capturing the consistency of the methods across simulations and case studies (Fig. 5d).

These two metrics were used to determine superior, satisfactory, and unsatisfactory methods according to the following cutoffs.

- Superior: in top 50% (3) of methods according to variance of log-ratio metrics.
- Satisfactory: not in top 50% (3) of methods according to variance of log-ratio metrics, but underperformed both BH and Storey’s *q*-value in less than 10% of case studies or simulation settings.
- Unsatisfactory: not in top 50% (3) of methods according to variance of log-ratio metrics, and underperformed both BH and Storey’s *q*-value in more than 10% of case studies or simulation settings.

#### Applicability

Ratings were determined using the proportion of case studies in which each method could be applied. The proportion was calculated first within each type of case studies, followed by averaging across all case studies. This was done to prevent certain types, e.g., scRNA-seq DE analysis, from dominating the average. The following cutoffs were used to determine superior, satisfactory, and unsatisfactory methods. For this metric, cases when adapt-glm returned exactly zero positive tests while all other methods returned non-zero results where labeled as data sets where the method could not be applied. This is denoted by an asterisk in Fig. 6.

- Superior: applied in 100% of case studies.
- Satisfactory: applied in more than 50% of case studies.
- Unsatisfactory: otherwise.

#### Usability

Ratings were determined based on our experience using the method for our benchmark comparison and rated according to the following criteria.

- Superior: a well-documented implementation is available.
- Satisfactory: an implementation is available, but lacks extensive documentation or requires additional work to install.
- Unsatisfactory: no implementation is readily available.

### Open Access

This article is distributed under the terms of the Creative Commons Attribution 4.0 International License (http://creativecommons.org/licenses/by/4.0/), which permits unrestricted use, distribution, and reproduction in any medium, provided you give appropriate credit to the original author(s) and the source, provide a link to the Creative Commons license, and indicate if changes were made. The Creative Commons Public Domain Dedication waiver (http://creativecommons.org/publicdomain/zero/1.0/) applies to the data made available in this article, unless otherwise stated.

### Additional files

Additional file 1 is available as supplementary material in *Genome Biology* and files 2-41 are available on GitHub at https://pkimes.github.io/benchmark-fdr-html/ [30].

## Additional files

Additional file 1 Supplementary Figures S1-S13, Supplementary Tables S1-S4, and Supplementary results. (PDF 981 kb)

Additional file 2: Analysis and benchmarking results under the null and using a unimodal alternative effect size distribution and large proportion (30%) of non-nulls using yeast RNA-seq data. (HTML kb)

Additional file 3: Analysis and benchmarking results using a unimodal alternative effect size distribution and small proportion (7.5%) of non-nulls using yeast RNA-seq data. (HTML kb)

Additional file 4: Analysis and benchmarking results using a bimodal alternative effect size distribution and large proportion (30%) of non-nulls using yeast RNA-seq data. (HTML kb)

Additional file 5: Analysis and benchmarking results using a bimodal alternative effect size distribution and small proportion (7.5%) of non-nulls using yeast RNA-seq data. (HTML kb)

Additional file 6: Analysis and benchmarking results using a bimodal alternative effect size distribution and large proportion (30%) of non-nulls using RNA-seq data simulated using Polyester. (HTML kb)

Additional file 7: Analysis and benchmarking results of simulation settings with only null tests, using normal, t, and χ2 distributed test statistics. (HTML kb)

Additional file 8: Analysis and benchmarking results of simulation settings with “cubic” informative covariate and normal, t, and χ2 distributed test statistics. (HTML kb)

Additional file 9: Analysis and benchmarking results of simulation settings with “step” informative covariate and normal, t, and χ2 distributed test statistics. (HTML kb)

Additional file 10: Analysis and benchmarking results of simulation settings with “sine” informative covariate and normal, t, and χ2 distributed test statistics. (HTML kb)

Additional file 11: Analysis and benchmarking results of simulation settings with “cosine” informative covariate and normal, t, and χ2 distributed test statistics. (HTML kb)

Additional file 12: Analysis and benchmarking results of simulation settings with “cubic” informative covariate, normal test statistics, and unimodal effect size distributions. (HTML kb)

Additional file 13: Analysis and benchmarking results of simulation settings with “cubic” informative covariate, t11 distributed test statistics, and unimodal effect size distributions. (HTML kb)

Additional file 14: Analysis and benchmarking results of simulation settings with “cubic” informative covariate, normal test statistics, unimodal effect size distributions, and higher (25% vs 10%) proportion of non-null tests. (HTML kb)

Additional file 15: Analysis and benchmarking results of simulation settings with “sine” informative covariate, normal test statistics, and varying total number of hypothesis tests. (HTML kb)

Additional file 16: Analysis and benchmarking results of simulation settings with “sine” informative covariate, normal test statistics, and varying proportion of null hypotheses. (HTML kb)

Additional file 17: Analysis and benchmarking results of simulation settings with “sine” informative covariate, t11 distributed test statistics, and varying proportion of null hypotheses. (HTML kb)

Additional file 18: Analysis and benchmarking results of simulation settings with informative covariates of varying informativeness using a continuous relationship between the covariate and the null proportion. (HTML kb)

Additional file 19: Analysis and benchmarking results of simulation settings with informative covariates of varying informativeness using a discrete relationship between the covariate and the null proportion. (HTML kb)

Additional file 20: Analysis and benchmarking results of simulation settings with “step” informative covariate comparing AdaPT with and without a null model option. (HTML kb)

Additional file 21: Analysis and benchmarking results of a meta-analysis of genome-wide association studies of body mass index. (HTML kb)

Additional file 22: Gene set analysis of differentially expressed mouse genes after HSC differentiation using goSeq. (HTML kb)

Additional file 23: Gene set analysis of differentially expressed human genes between the cerebellum and cerebral cortex using goSeq. (HTML kb)

Additional file 24: Gene set analysis and benchmarking results of differentially expressed mouse genes after HSC differentiation using GSEA. (HTML kb)

Additional file 25: Gene set analysis and benchmarking results of differentially expressed human genes between the cerebellum and cerebral cortex using GSEA. (HTML kb)

Additional file 26: Differential expression analysis and benchmarking results of human genes between nucleus accumbens and putamen. (HTML kb)

Additional file 27: Differential expression analysis and benchmarking results of human genes between microRNA knockdown and control mouse cells. (HTML kb)

Additional file 28: Single-cell differential expression analysis and benchmarking results between human glioblastoma tumor cells and nearby controls using MAST. (HTML kb)

Additional file 29: Single-cell differential expression analysis and benchmarking results between human glioblastoma tumor cells and nearby controls using scDD. (HTML kb)

Additional file 30: Single-cell differential expression analysis and benchmarking results between human glioblastoma tumor cells and nearby controls using Wilcox. (HTML kb)

Additional file 31: Single-cell differential expression analysis and benchmarking results between stimulated murine macrophage cells and controls using MAST. (HTML kb)

Additional file 32: Single-cell differential expression analysis and benchmarking results between stimulated murine macrophage cells and controls using scDD. (HTML kb)

Additional file 33: Single-cell differential expression analysis and benchmarking results between stimulated murine macrophage cells and controls using Wilcox. (HTML kb)

Additional file 34: Differential binding analysis and benchmarking results of H3K4me3 between two cell lines in promoter regions using DESeq2. (HTML kb)

Additional file 35: Differential binding analysis and benchmarking results of H3K4me3 between two cell lines using csaw. (HTML kb)

Additional file 36: Differential binding analysis and benchmarking results of CREB-binding protein between knockout and wild-type mice using csaw. (HTML kb)

Additional file 37: Differential abundance analysis and benchmarking results of obesity. (HTML kb)

Additional file 38: Differential abundance analysis and benchmarking results of IBD. (HTML kb)

Additional file 39: Differential abundance analysis and benchmarking results of infectious diarrhea. (HTML kb)

Additional file 40: Differential abundance analysis and benchmarking results of colorectal cancer. (HTML kb)

Additional file 41: Correlation analysis and benchmarking results of wastewater contaminants. (HTML kb)
