# Supplementary material for: A practical guide to methods controlling false discoveries in computational biology
Source: Genome Biol. 2019 Jun 4;20:118. doi: 10.1186/s13059-019-1716-1 (PMC6547503; doi:10.1186/s13059-019-1716-1)
Supplement: Supplementary file 1 — Supplementary Figures S1-S13, Supplementary Tables S1-S4, and Supplementary results. (PDF 981 kb) [file 13059_2019_1716_MOESM1_ESM.pdf]

Additional file 1
Supplementary Figures and Tables

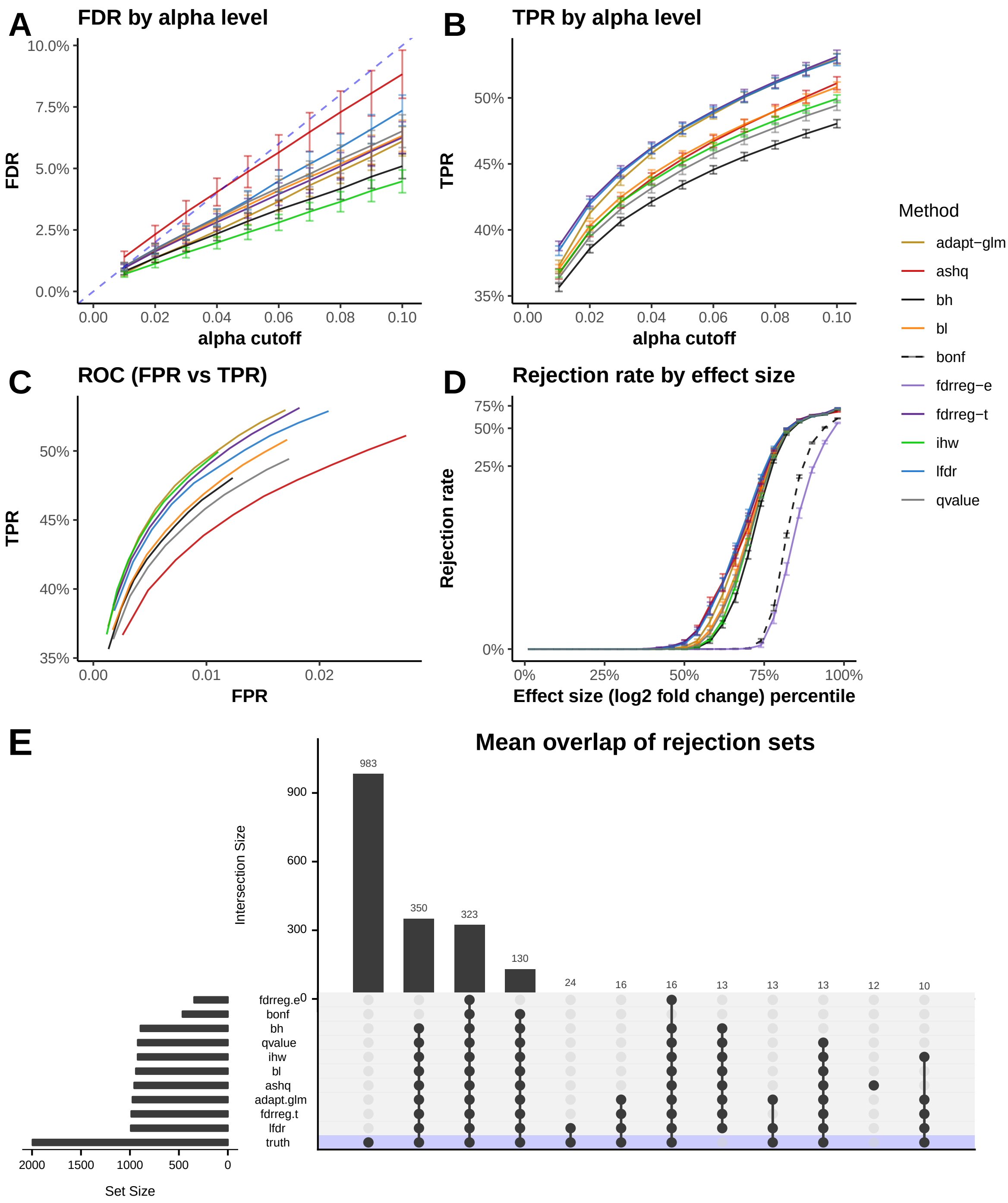

Figure S1 Performance evaluation in yeast *in silico* experiment. Using RNA-seq data derived from a yeast experiment, a differential expression analysis was performed comparing two groups with five samples each and differential signal added to 2000 out of approximately 6500 genes (30%) using an informative covariate. An evaluation of the performance includes the (A) FDR, (B) TPR, and (C) number of rejections by FDR (alpha) cutoff. (D) Percentage significant at 0.05 cutoff (log-scale) by effect size (log2 fold change) percentile. Method is denoted by color and the mean value across 100 replications with standard error bars is shown in (A)-(D). (E) Mean overlap of discoveries at 0.05 cutoff across methods and underlying truth.

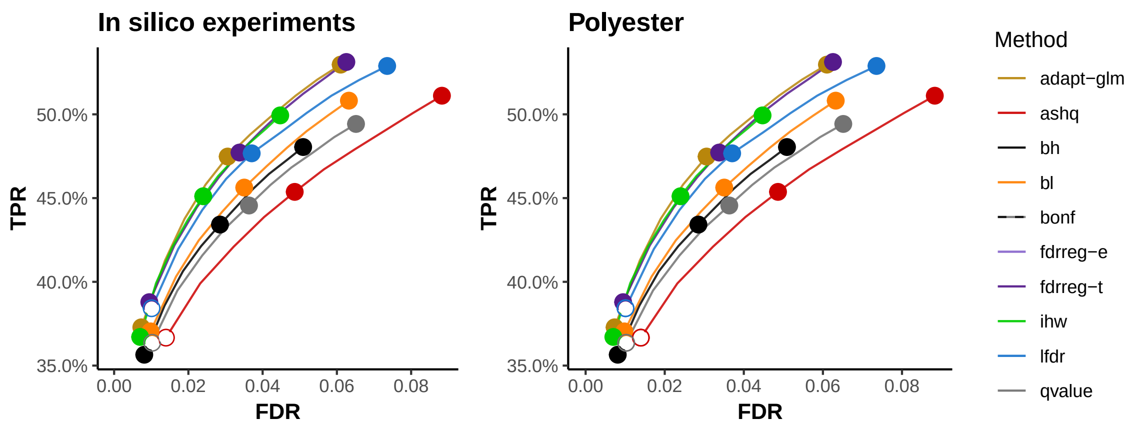

Figure S2 FDR versus TPR in *in silico* experiments and simulations. Following the style of the R/Bioconductor iCOBRA package [1], the average FDR is plotted on the x-axis against the average TPR on the y-axis for the yeast RNA-seq *in silico* resampling experiments (left) and RNA-seq counts using the `polyester` R/Bioconductor package (right). Three points are included for each line (method) at the following nominal α values: 0.01, 0.05, and 0.10. A solid point indicates FDR was controlled at the nominal level (on average), whereas an open point indicates that it was not. Averages are taken over all 100 simulation replications.

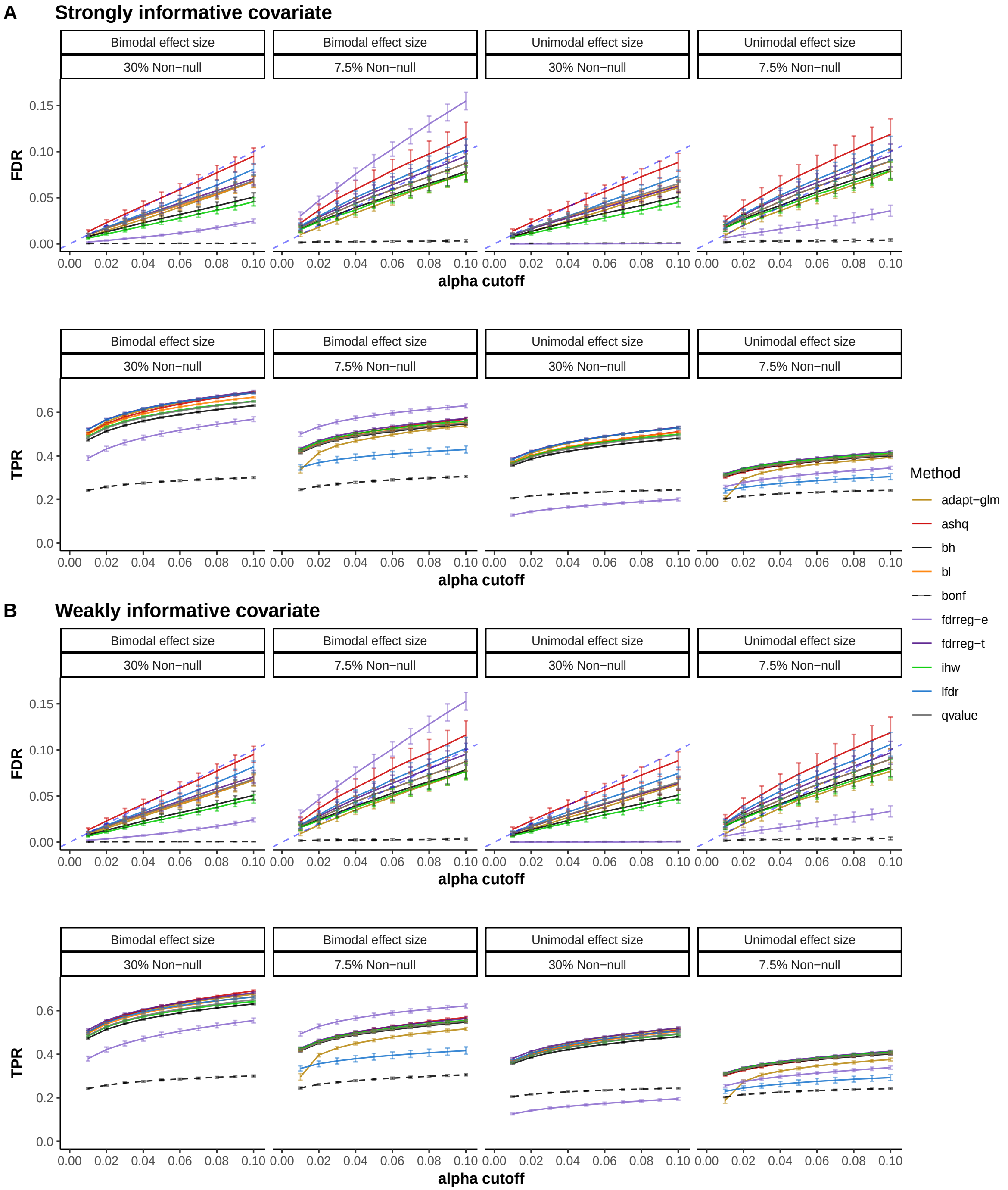

Figure S3 FDR and TPR under various spike-in settings of yeast *in silico* experiments. Plots of FDR and TPR across α cutoff values over 100 replications in the yeast simulation (sample size 5 in each group) by alpha level. Vertical bars depict standard error. Each panel within A and B represents a combination of settings for π_0: 30% (2000) and 7.5% (500) non-null genes (total of 6500 genes), as well as different non-null effect size distributions: bimodal and unimodal. (A) For a *strongly informative covariate*: the informative covariate is equal to the sampling weights for non-null genes. (B) For a *weakly informative covariate*: the informative covariate is equal to the sampling weights for selecting non-null genes plus noise.

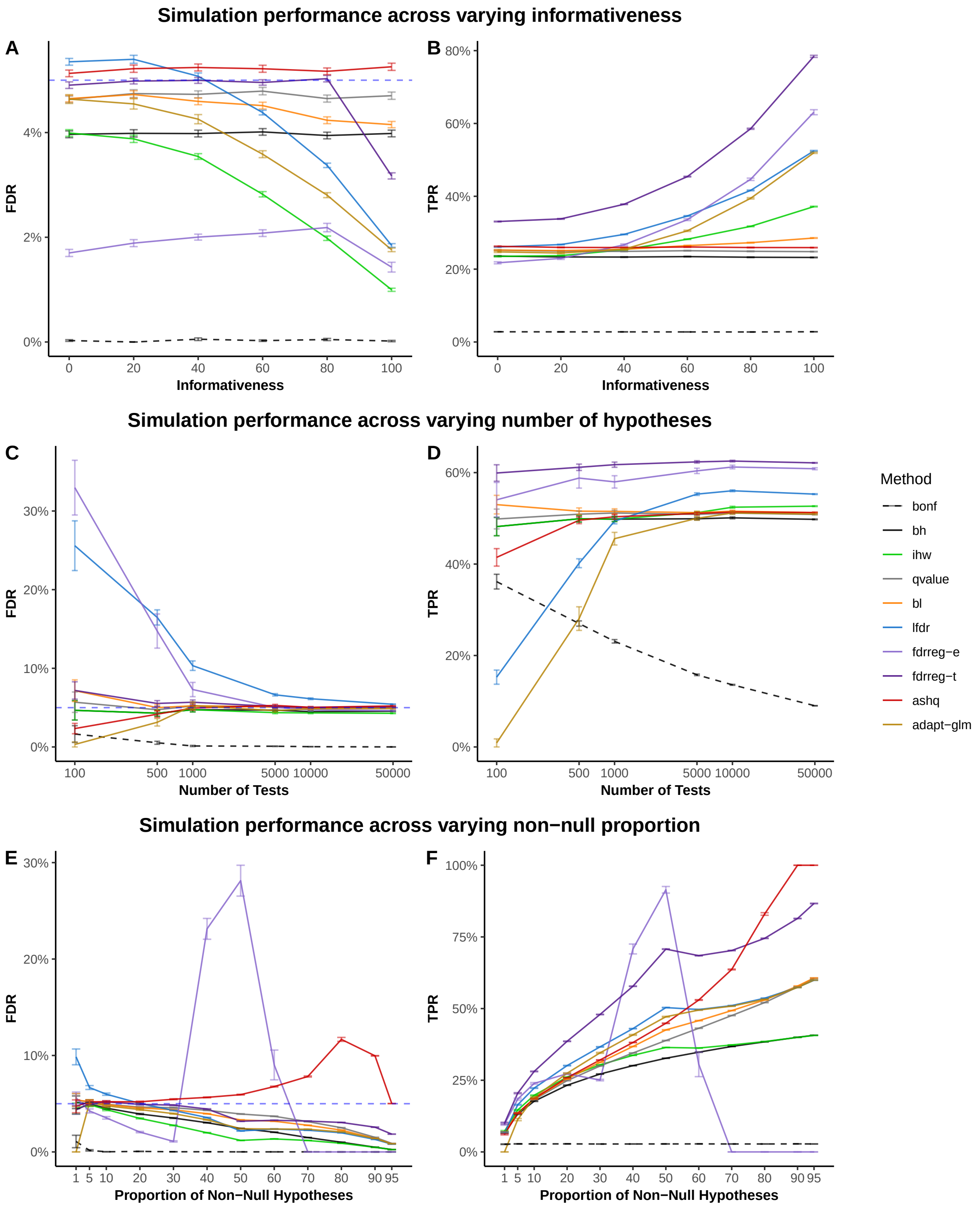

Figure S4 Performance evaluation in simulations. (A) FDR control and (B) TPR across varying informativeness. (C) FDR and (D) TPR across varying number of hypotheses (E) FDR and (F) TPR across varying proportions of null and alternative hypotheses. Method is denoted by color and the mean value across 100 replications with standard error bars is shown in (A)-(F). The FDR and TPR are shown at the nominal 0.05 cutoff.

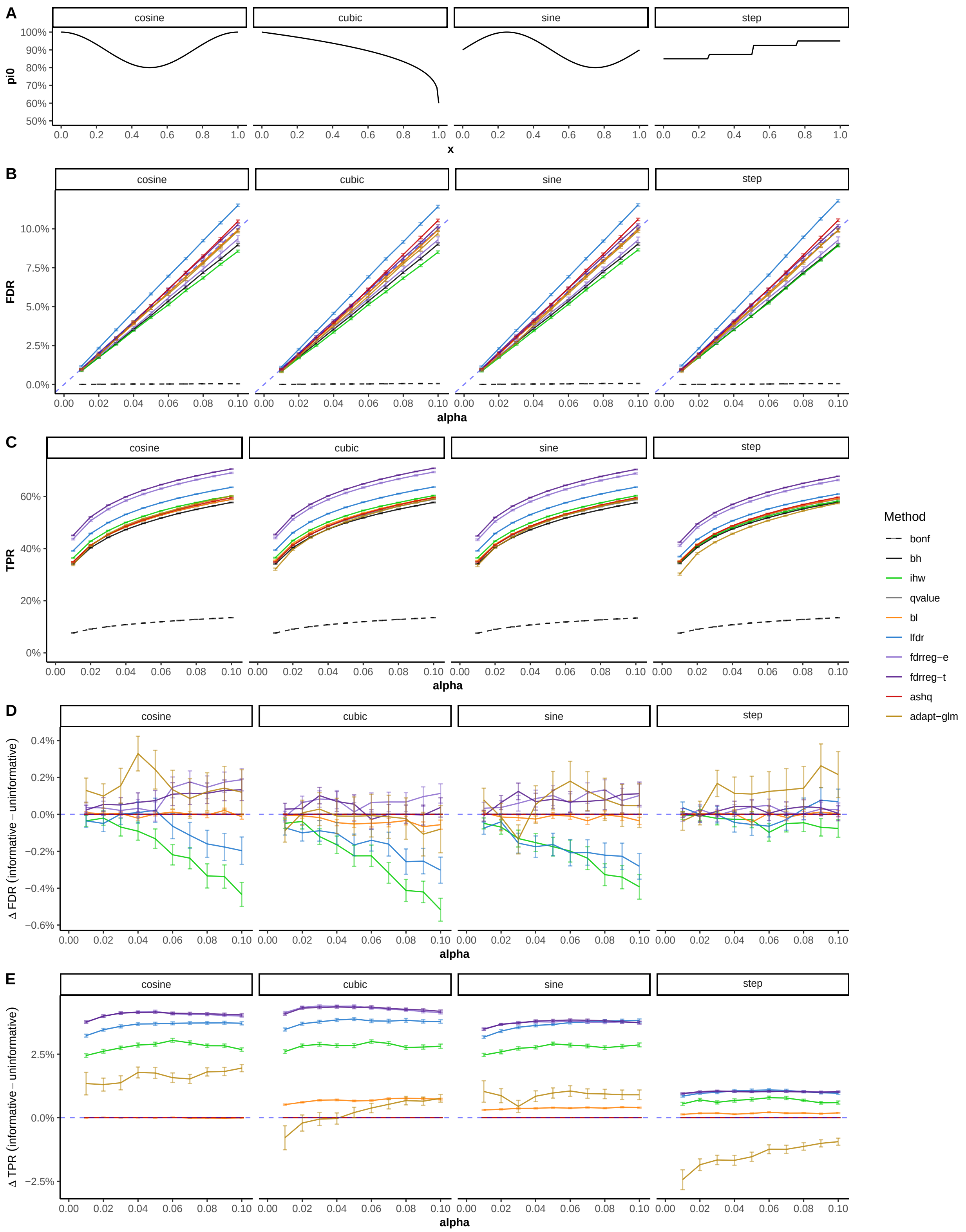

Figure S5 Simulation performance across informative covariate relationship (A) Relationship between covariate value, x, and null proportion of hypotheses, π_0, across simulation settings. (B) FDR and (C) TPR across nominal FDR thresholds between 0.01 and 0.10 are shown for each method across four informative covariates described in the "Methods" section. Differences in (D) FDR and (E) TPR between informative and uninformative covariates are also shown.

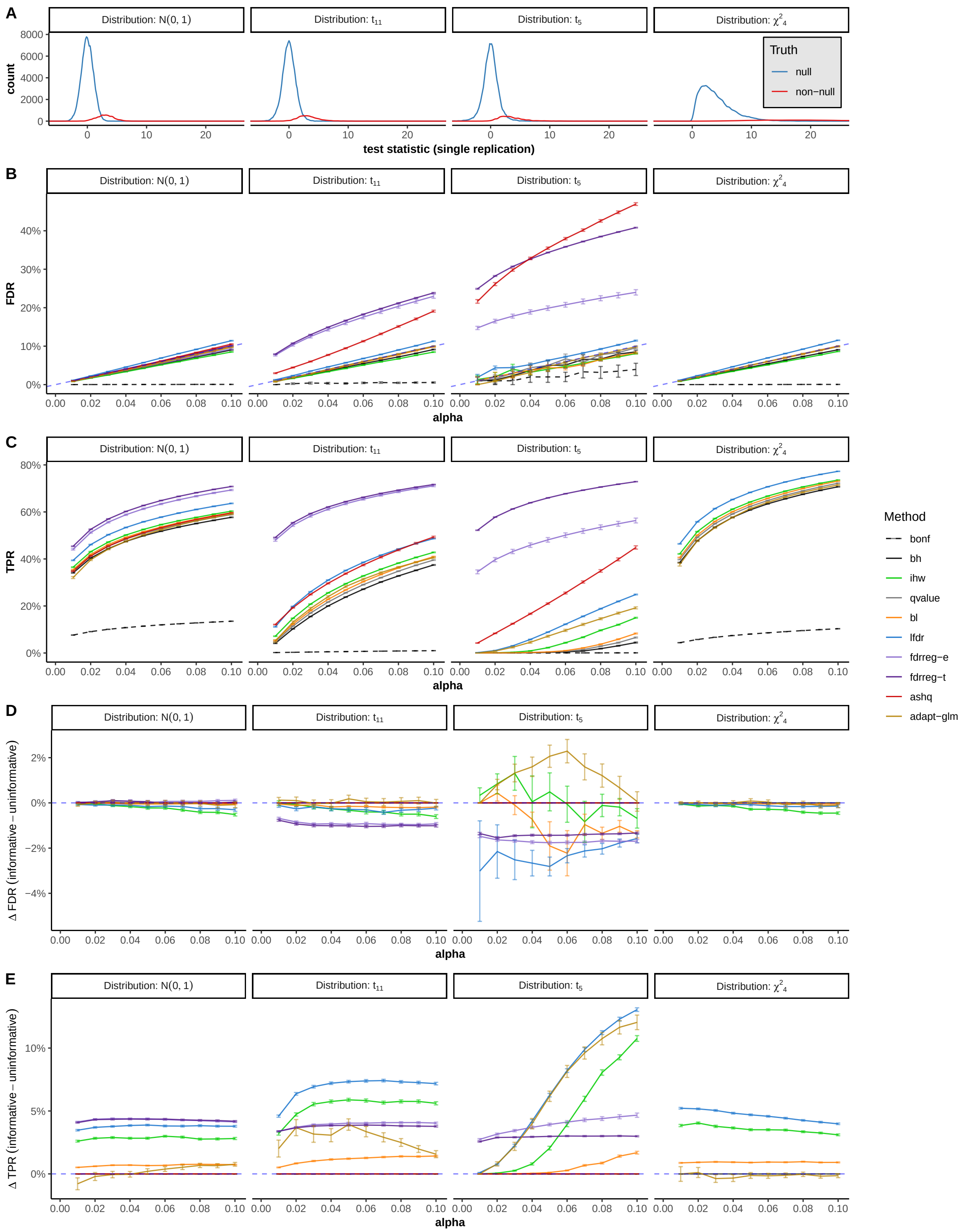

Figure S6 Simulation performance across test statistic distributions (A) Distributions of test statistics for null and non-null tests in one replication of each simulation. (B) FDR control and (C) TPR across different test statistic distributions. Differences in (D) FDR and (E) TPR between informative and uninformative covariates are also shown. Method is denoted by color and the mean value across 100 replications with standard error bars is shown in (B)-(E). Results for ash, fdrreg-t, fdrreg-e are not shown for the χ^2_4 setting due to due to distributional assumptions of the methods (see Figure 1). Method is denoted by color and the mean value across 100 replications with standard error bars is shown in (B)-(E).

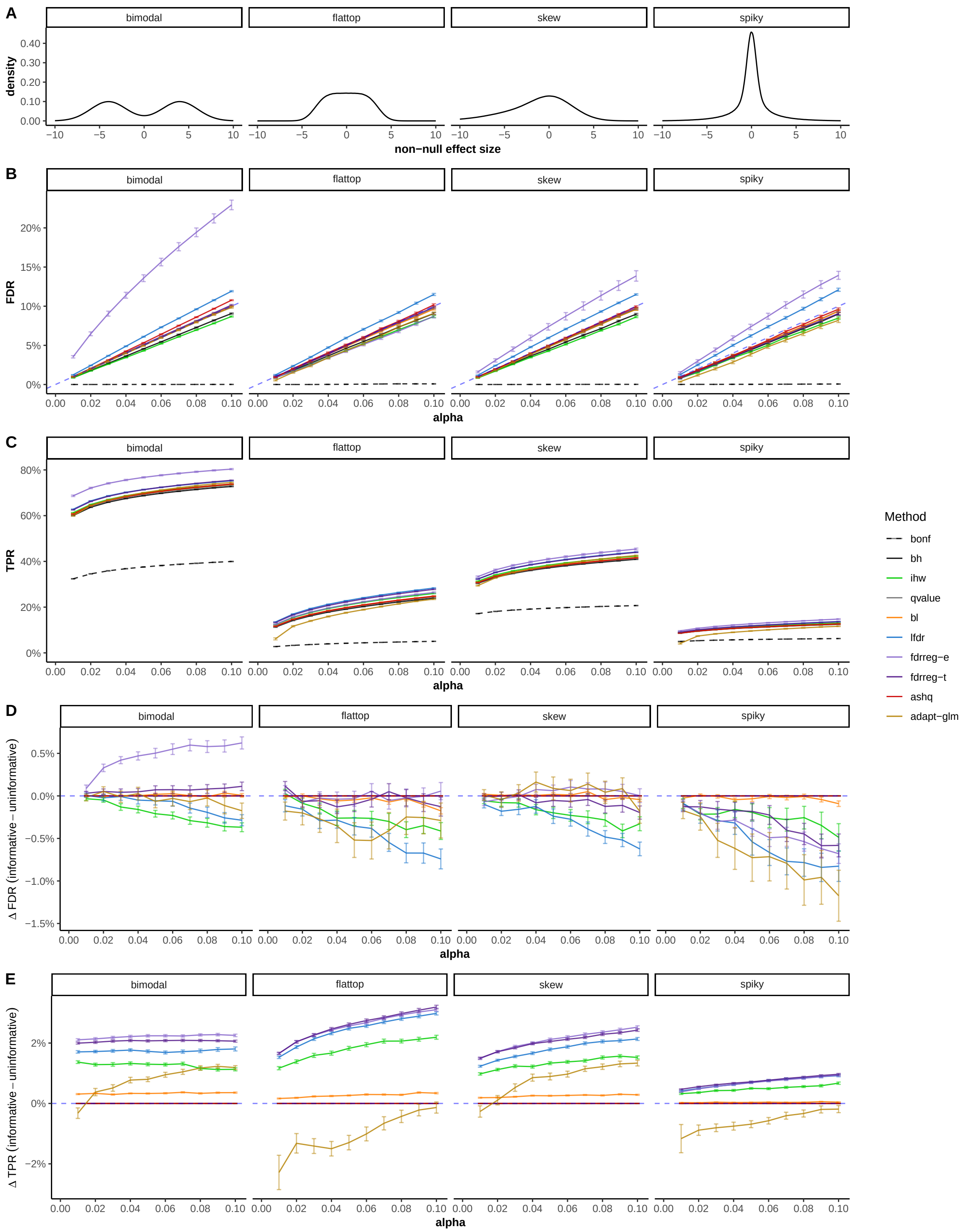

Figure S7 Simulation performance across effect size distributions (A) Distributions of effect sizes included in unimodal effect size simulations. (B) FDR and (C) TPR across nominal FDR thresholds between 0.01 and 0.10 are shown for each method across four distributions of the non-null effect sizes presented in [2]: bimodal, flat-top, skew, and spiky. All distributions are unimodal with mode at zero except for the "bimodal" setting. Settings are tested to evaluate the performance of ASH against all other methods under the unimodal assumption. Differences in (D) FDR and (E) TPR between informative and uninformative covariates are also shown. Method is denoted by color and the mean value across 100 replications with standard error bars is shown in (B)-(E).

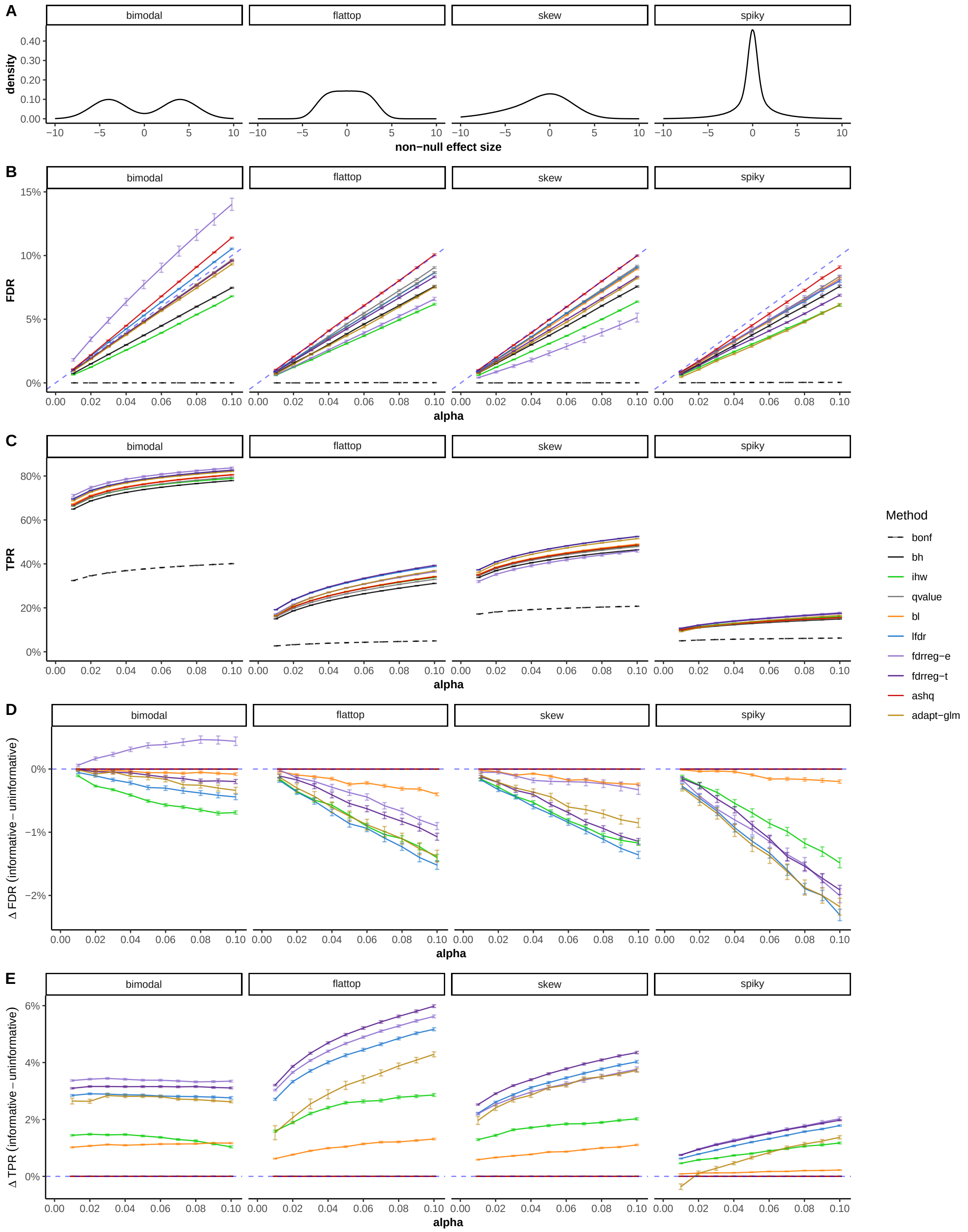

Figure S8 Simulation performance across unimodal effect size distributions w/ 25% non-null Same as Figure S7 but with increased proportion of non-null hypotheses. (A) Distributions of effect sizes included in unimodal effect size simulations. (B) FDR and (C) TPR across nominal FDR thresholds between 0.01 and 0.10 are shown for each method across four distributions of the non-null effect sizes. Differences in (D) FDR and (E) TPR between informative and uninformative covariates are also shown. Method is denoted by color and the mean value across 100 replications with standard error bars is shown in (B)-(E).

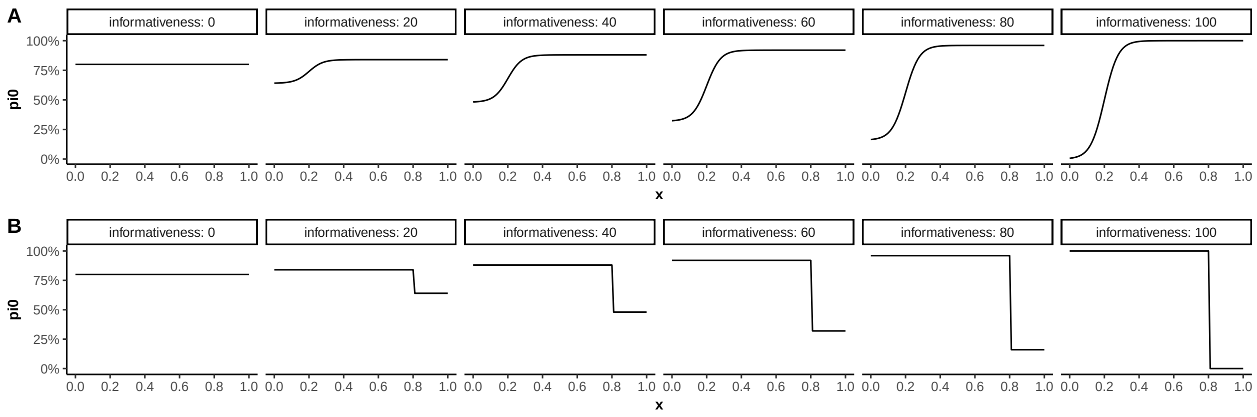

Figure S9 Informativeness covariate relationships. Relationship between covariate value, x, and null proportion of hypotheses, π_0, across several δ informativeness values for (A) $p^{\text{c-info}}$ and (B) $p^{\text{s-info}}$.

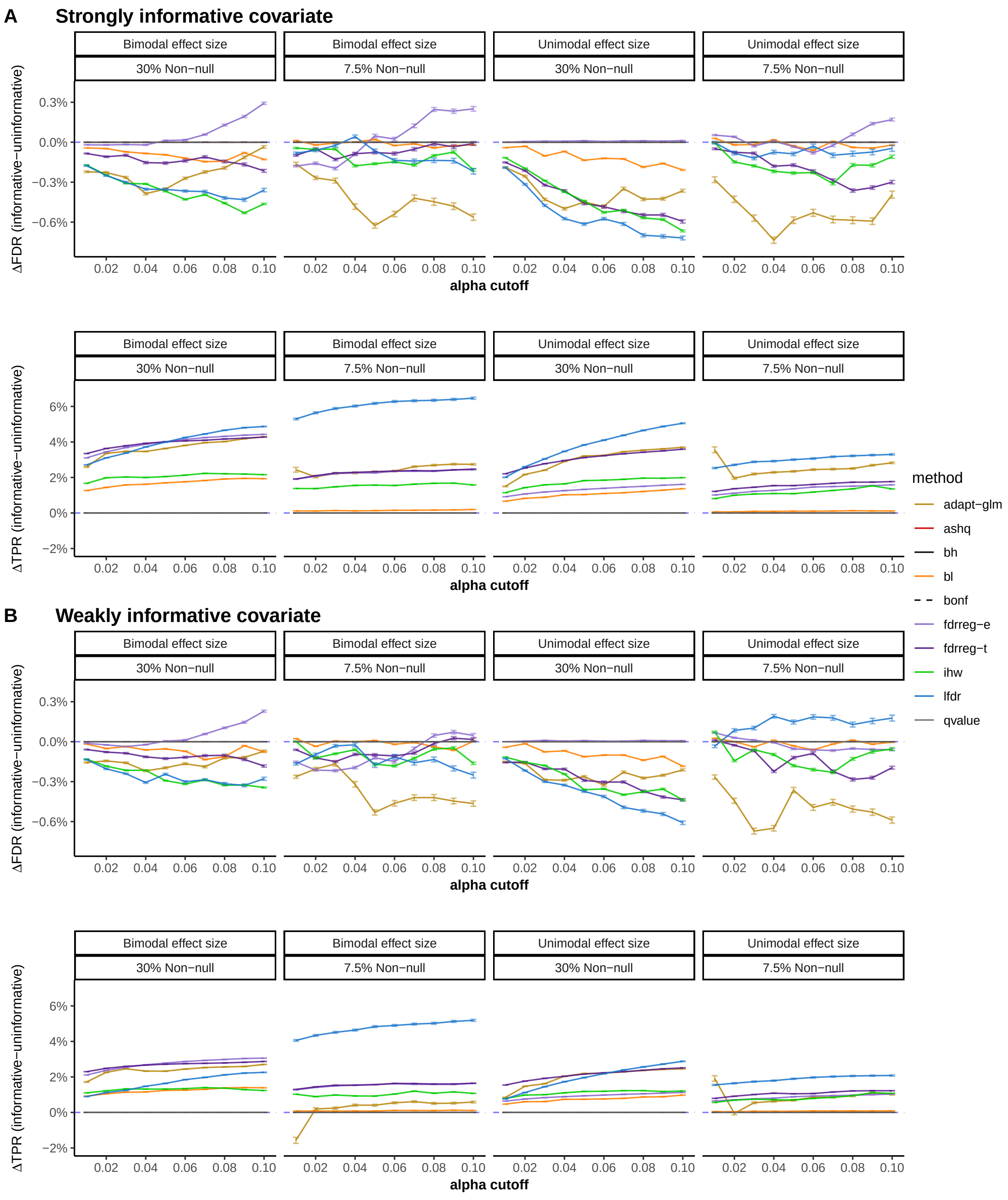

Figure S10 Impact of covariate information in yeast experiments Mean difference of FDR and TPR (with informative covariate - without informative covariate) over 100 replications in the yeast simulation (sample size 5 in each group) by alpha level. Vertical bars depict standard error. Each panel within A and B represents a combination of settings for π_0: 30% (2000) and 7.5% (500) non-null genes (total of 6500 genes), as well as different non-null effect size distributions: bimodal and unimodal. (A) For a *strongly informative covariate*: the informative covariate is equal to the sampling weights for non-null genes, and a (B) For a *weakly informative covariate*: the informative covariate is equal to the sampling weights for selecting non-null genes plus noise.

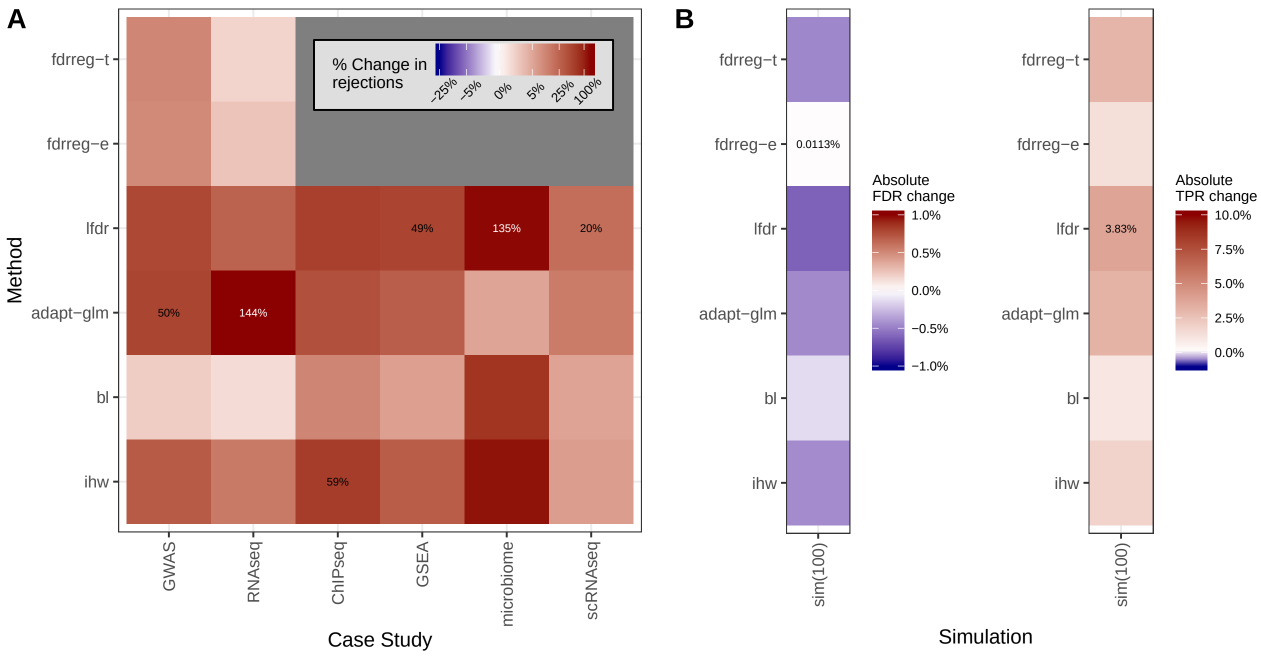

Figure S11 Gain from informative covariate varies by case study. (A) For each method (y-axis) that uses an informative covariate, the percent change in rejections when using an informative covariate as compared to using a completely uninformative covariate is represented by color. This is defined as number of rejections when using the informative covariate divided by the number of rejections when using the uninformative (random) covariate, multiplied by 100. This value is averaged across all datasets and informative covariates used in each case study (x-axis). If rejections were found using the informative covariate but none were found using the uninformative covariate, the percentage was set to 100%. The maximum value of this percentage in each column is labeled. (B) For each method (y-axis) that uses an informative covariate, the absolute percentage change in FDR (left) and TPR (right) in the yeast *in silico* experiments are represented in color (setting with 5 samples in each group, unimodal effect size distribution, and 30% non-null genes). Results are averaged over 100 simulation replications.

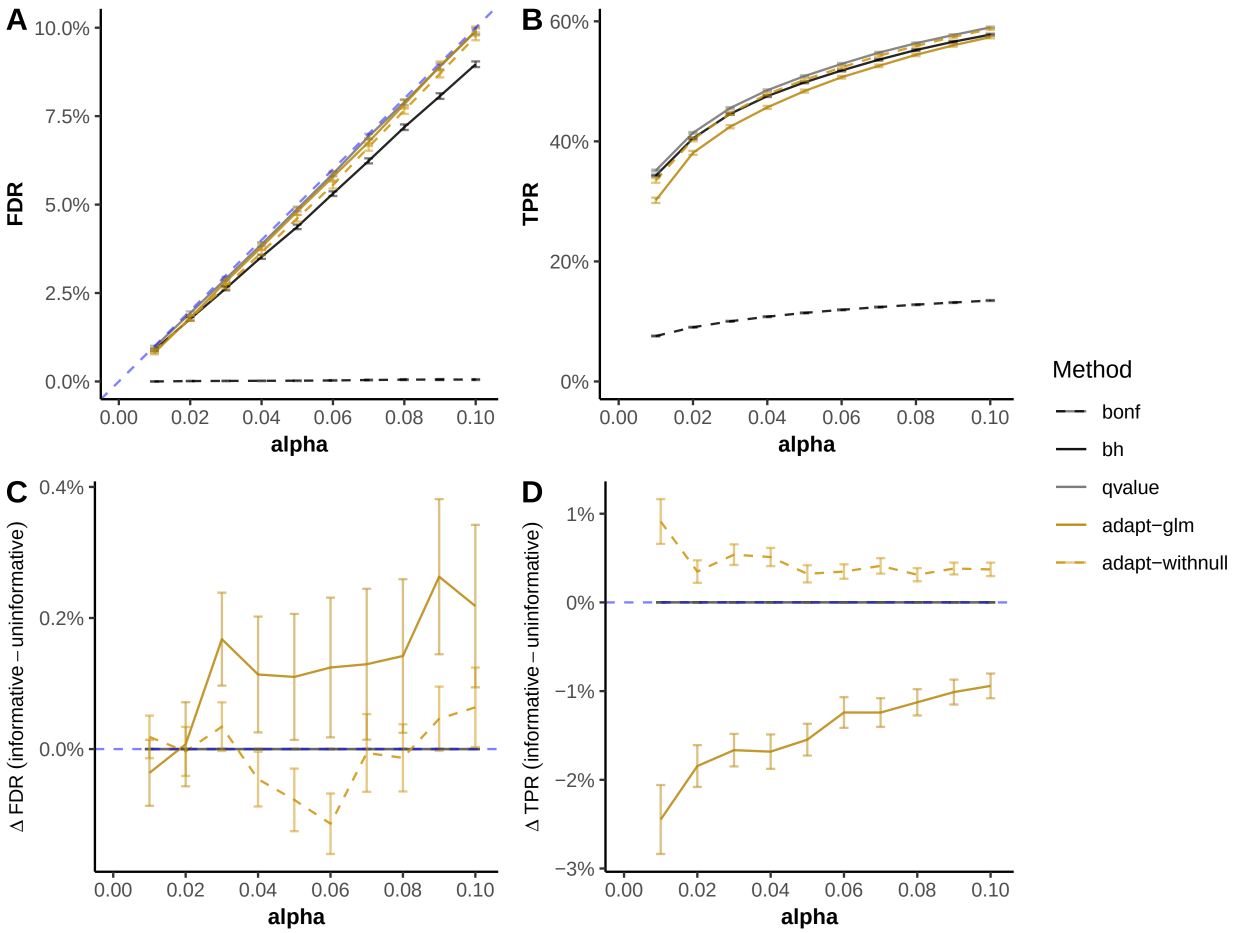

Figure S12 Simulation performance with modified AdaPT parameters (A) FDR and (B) TPR across nominal FDR thresholds between 0.01 and 0.10 are shown for a subset of methods with the "step" informative covariate (Figure S5). Differences in (C) FDR and (D) TPR between informative and uninformative covariates are also shown. AdaPT with model parameters modified to include a null model is shown as "adapt-withnull" and compared against the default AdaPT call, adapt-glm. Method is denoted by color and the mean value across 100 replications with standard error bars is shown in (A)-(D).

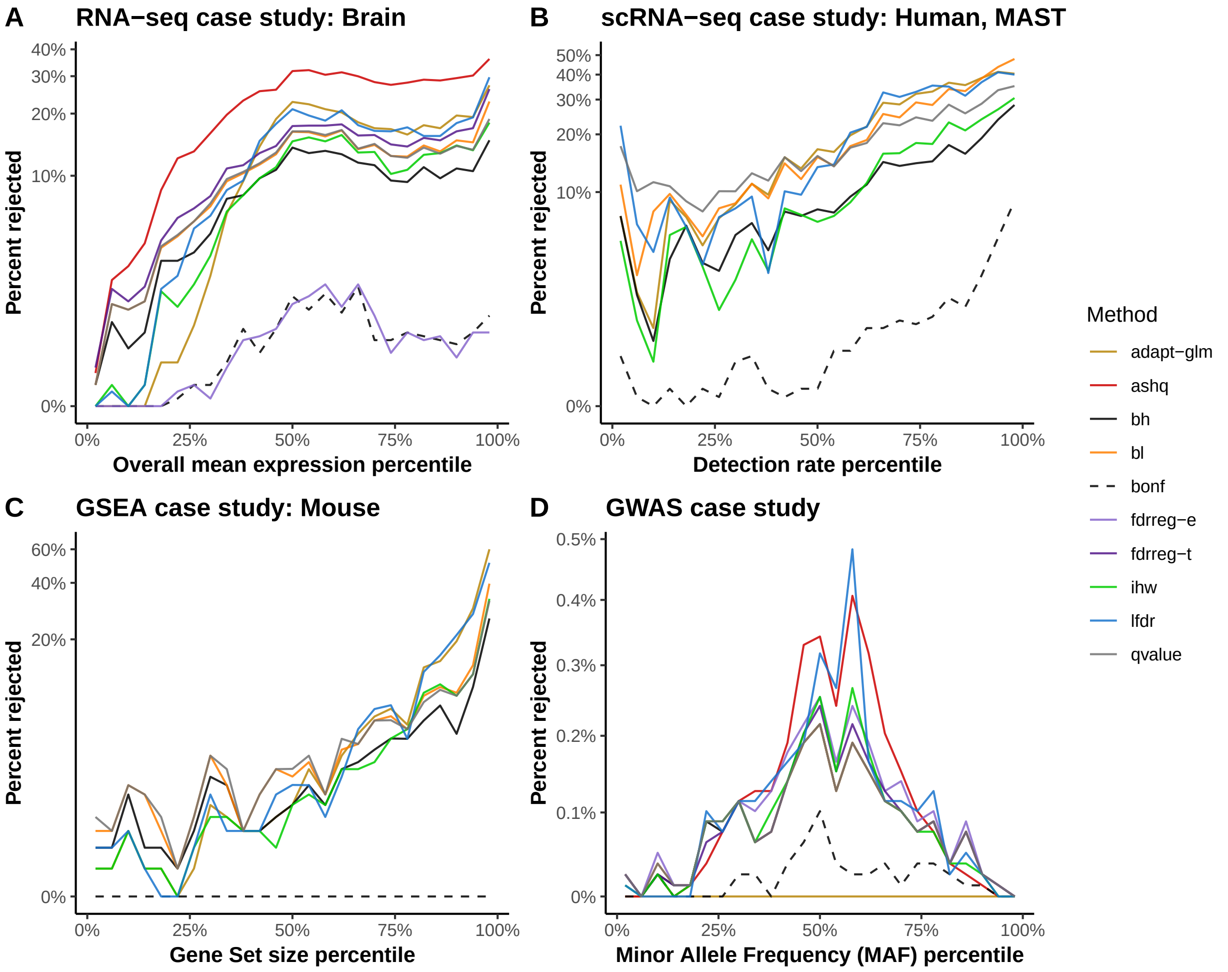

Figure S13 Relationship between informative covariate and rejection rate was highly variable across case studies. Four informative covariates from four different case study datasets were chosen to illustrate the wide variation in the relationship between the informative covariate and the proportion of tests rejected across all FDR controlling methods. (A) Relationship between the overall mean expression percentile and rejection rate in the brain RNA-seq dataset. (B) Relationship between the detection rate percentile and the rejection rate in the Human single cell RNA-seq dataset. (C) Relationship between the gene set size percentile and the rejection rate in the Mouse GSEA dataset. (D) Relationship between the minor allele frequency (MAF) percentile and the rejection rate in the GWAS case study.

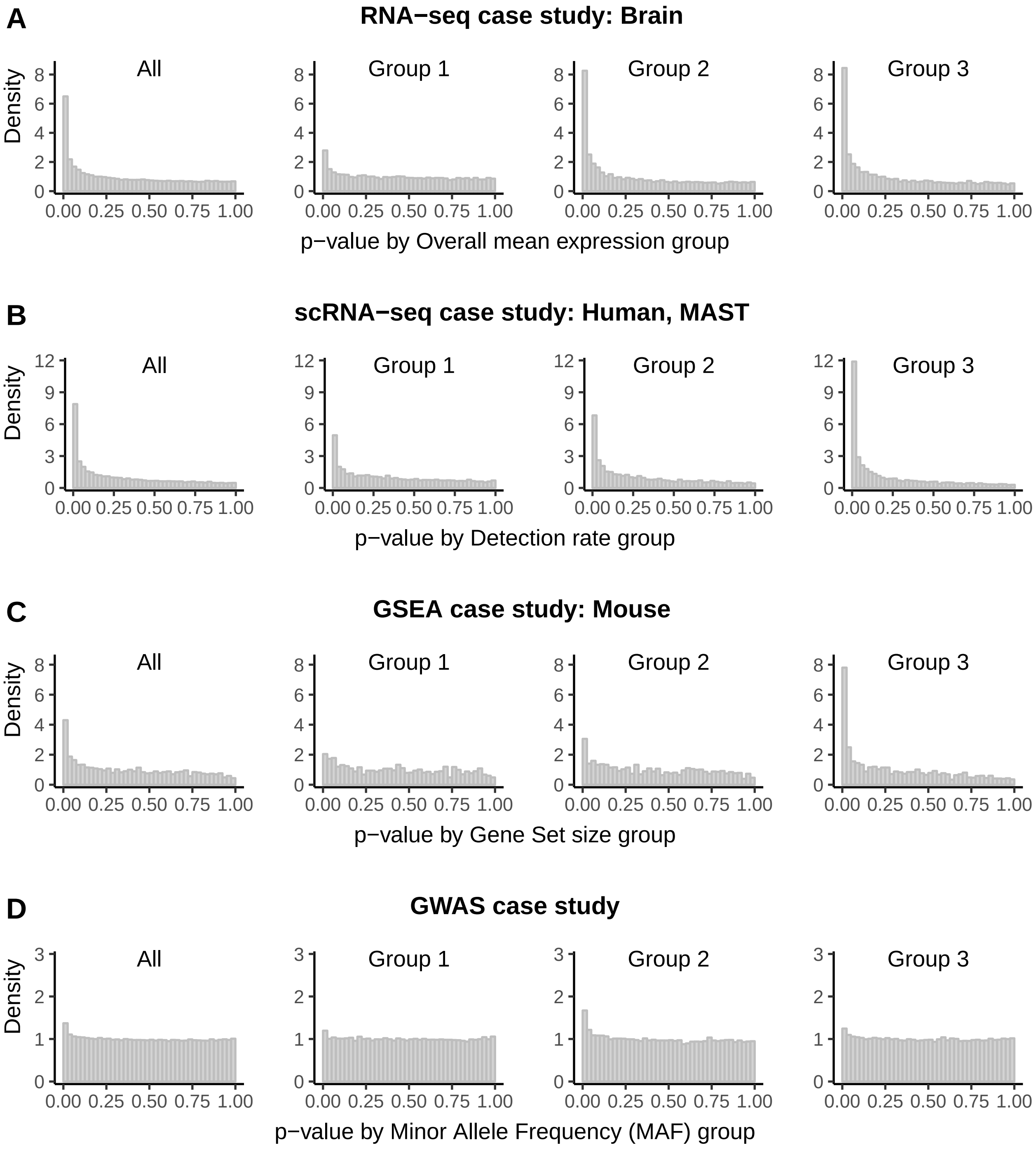

Figure S14 Examples of informative covariates that are independent under the null hypothesis. Distribution of p-values overall (left-most panel) and in three approximately equal sized bins by informative covariate (right panels) for (A) the brain dataset in the RNA-seq case study using overall mean expression as the informative covariate, (B) the human dataset in the single-cell RNA-seq case study analyzed by MAST using the detection rate as the informative covariate, (C) the mouse dataset of the GSEA case study using the gene set size as the informative covariate, and (D) the GWAS case study using the minor allele frequency as the informative covariate.

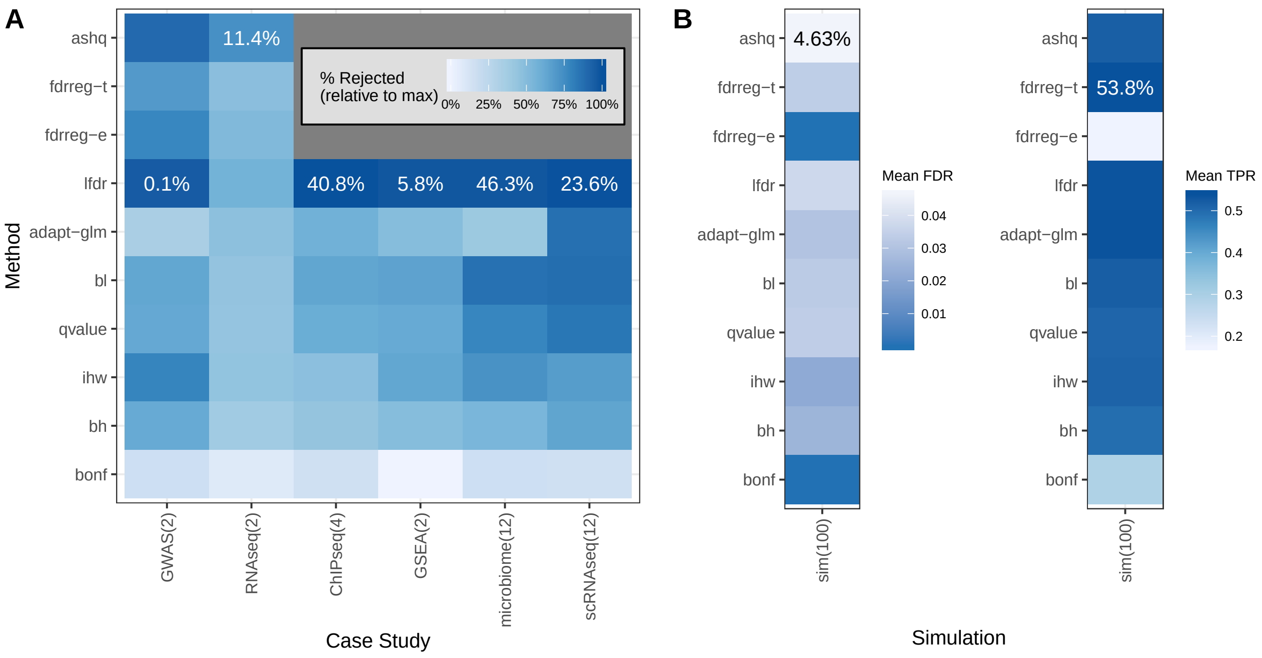

Figure S15 Case study evaluation (A) Mean proportion of maximum rejections (color) across all datasets and informative covariates used for each case study (column) and FDR correction method (row). In each column, the maximum proportion of rejections out of the total possible number of comparisons is displayed. (B) Mean FDR and TPR (values displayed in each tile and represented by color) in yeast simulation study at target FDR level 0.05 across sample size settings and 100 replications for each FDR correction method (row). Numerical values for the data in (A) are displayed in Table S4.

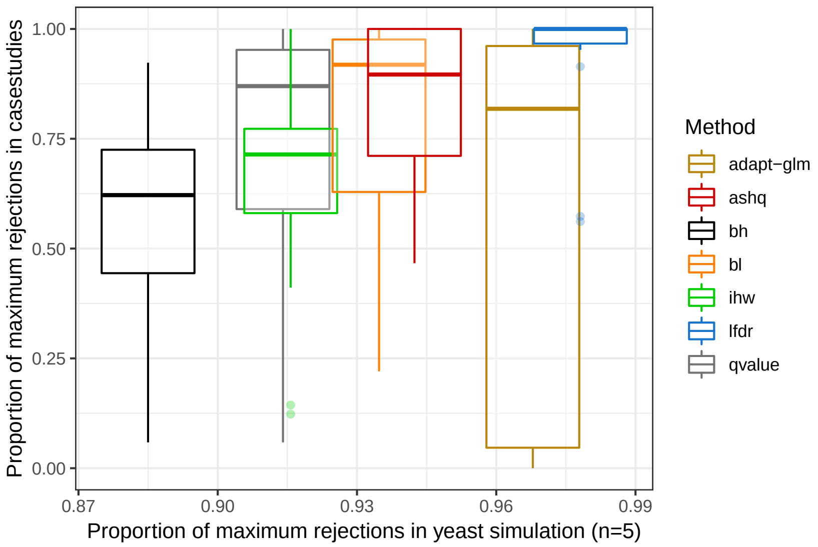

Figure S16 Comparison of the number of rejections in yeast simulation and case studies. Boxplots of the proportion of maximum rejections across all case studies (y-axis) is shown for each method, where the x-axis position reflects the proportion of maximum rejections in the yeast simulation (setting with sample size 5 in each group, unimodal effect size distribution, and 30% non-null genes). The alpha cutoff to determine rejections is 0.05.

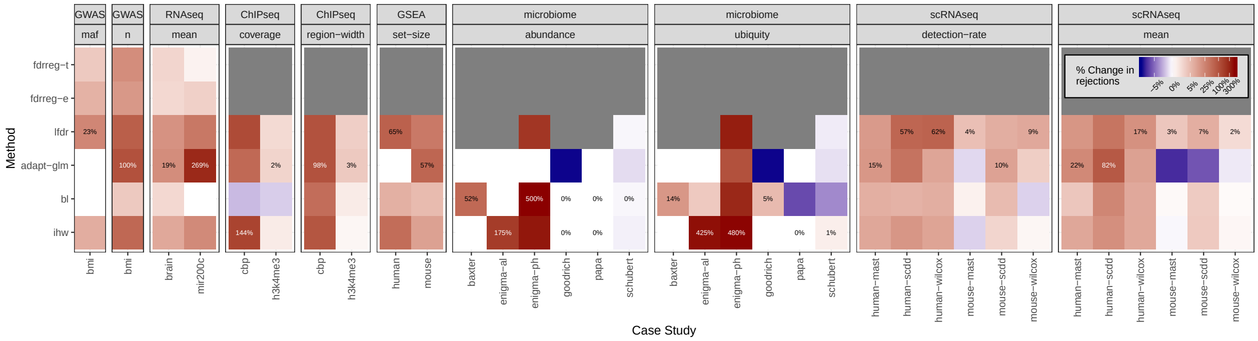

Figure S17 Gain from informative covariate varies by dataset and covariate in case studies. For each method (y-axis) that uses an informative covariate, the percent change in rejections when using an informative covariate as compared to using a completely uninformative covariate is represented by color. This is defined as number of rejections when using the informative covariate divided by the number of rejections when using the uninformative (random) covariate, multiplied by 100. This is shown separately for each dataset (grouped by case study and informative covariate, x-axis). If rejections were found using the informative covariate but none were found using the uninformative covariate, the percentage was set to 100%. The maximum value of this percentage in each column is labeled. The informative covariate used in each case study is listed in Table 1. For case studies with more than one covariate, the covariate is denoted in the column labels.

Table S1 Approaches to adjust for multiple comparisons across hypothesis tests. The family-wise error rate (FWER) is the probability of at least one false discovery. The false discovery rate (FDR) is the expected fraction of false discoveries among all discoveries. FDR adjusted p-values are defined as adjusted p-values that have control FDR at nominal Type 1 error (α) level. π_0 represents the proportion of null hypothesis tests.

Control	Method	Input	Output	Two Groups Model $x_i \sim \pi_{0,i} * f_{0,i} + (1 - \pi_{0,i}) * f_{1,i}$				Description	Availability (R)
				$x_i =$	$\pi_{0,i} =$	$f_{0,i} =$	$f_{1,i} =$		
FWER	Bonferroni correction [3, 4]	(1) p-values	adjusted p-values[1]	-	-	-	-	Robust to dependence, but very conservative	package: stats function(s): p.adjust
FDR	Benjamini-Hochberg Procedure (BH) [5]			p-value	equal across tests	equal across tests	equal across tests	First method proposed for controlling the FDR. More powerful than controlling FWER. However, previously shown to have sub-optimal power when the individual tests differ in statistical properties such as sample size, true effect size, signal-to-noise ratio or prior probability of being false [6].	package: stats function(s): p.adjust
	Storey's q-value [7]		q-value[2]	test statistic				Directly estimates π_0. Increasingly more powerful than BH as π_0 decreases, while controlling FDR.	package: qvalue function(s): qvalue
	Independent Hypothesis Weighting (IHW) [6]	(1) p-values, (2) independent covariate	adjusted p-values	p-value	equal within covariate groups	equal across tests	equal within covariate groups	Weighted BH method to prioritize tests using data-derived weights computed for groups of tests binned according to an independent covariate. Does not calculate $\pi_{0,i}$ explicitly.	package: ihw function(s): ihw, adjust_pvalues
	Boca and Leek Procedure (BL) [8]			test statistic	continuous (logistic) function of covariate	equal across tests	equal across tests	Weighted BH method to prioritize tests using data-derived weights computed using logistic regression with the independent covariate. Equivalent to Storey's q-value in the case of no covariates.	package: swfdr function(s): lm_pi0 [3]
	Cai and Sun's Conditional Local FDR (lfdr) [9]				equal within covariate groups	equal within covariate groups	equal within covariate groups	Modifies the standard two-group model by assuming known group structure and using different cutoffs for each group. FDR is controlled at different rates for each group to minimize the global false nondiscovery rate (FNR) subject to a constraint on the global FDR.	none [4]
	AdaPT (adapt-glm) [10]		q-values[5]	p-value	continuous (logistic) function of covariate	equal across tests	continuous density (exponential family), with parameter modeled as a continuous function (glm) of covariate	Modifies the Barber-Candès procedure [11, 12][6] by introducing an iterative, data-adaptive thresholding algorithm. The threshold depends on the covariate through the estimates of $\pi_{0,i}$ and $f_{1,i}$.	package: adaptMT function(s): adapt, adapt_glm, adapt_gam or adapt_glmnet [7]
	FDR Regression (FDRreg) (empirical) [13]	(1) z-scores, (2) independent covariate	Bayesian FDRs	test statistic	continuous (logistic) function of covariate	equal across tests	equal across tests	Modifies the standard two-group model by modeling the mixing proportions of the distributions for each test as a logistic function of an informative covariate (or spline expansion of the covariate). Assumes that the test statistics are normally distributed, with arbitrary mean and variance (empirical null) or standard normal (theoretical null).	package: FDRreg function(s): FDRreg [8]
	FDR Regression (FDRreg) (theoretical) [13]								
	Adaptive Shrinkage (ASH) [2]	(1) effect sizes, (2) standard error	q-values[9]	effect size	equal across tests	equal across tests	inversely proportional to power of standard error	Introduces the concept of the local false sign rate and s-values for controlling errors across multiple tests. Same approach can also be used to compute q-values and the local false discovery rate. Assumes that the distribution of effect sizes is unimodal.	package: ashr function(s): ash, get_qvalue

[1]Formally, the BH approach does not generate adjusted p-values, but instead provides significance calls at a specified α FDR cutoff. Adjusted p-values are commonly computed as the smallest FDR cutoffs at which each test would be called significant.

[2]The q-value is defined as the positive FDR (pFDR) analogue of the p-value. Approach can also be used to compute the local false discovery rate.

[3]Requires specification of a degrees of freedom parameter. Requires multiplying weights against BH adjusted p-values to obtain adjusted p-values

[4]Custom implementation using fdrtools package (function: fdrtools). Requires manually specifying covariate groups, computing local fdr with fdrtools package, followed by custom code

[5]q-value is defined as the minimum target FDR level such that the p-value is rejected. For hypotheses with p-values above the initial threshold value (default 0.45), the q-values are set to ∞ because they are never rejected by adapt-glm for any α.

[6]The Barber-Candès procedure estimates the false discovery proportion by quantifying the asymmetry in the distribution of p-values at a given threshold.

[7]adapt_glm, adapt_gam, and adapt_glmnet are all wrappers around adapt that encode a specific assumption about the relationship between $\pi(x)$ and $\mu(x)$. Requires manually specifying the models for these relationships with, e.g. splines package.

[8]Requires manually specifying model matrix with, e.g. splines package

[9]The method also returns local false sign rates, local false discovery rates, and s-values, where s-vales are defined analogous to Storey's q-value, but with the local false sign rate rather than the local false discovery rate. Since the aim of our analysis was to compare methods for controlling the FDR, we only report results for the estimated q-values.

Table S2 Yeast *in silico* experiment settings. The results from each series of simulations is reported as a separate Additional file in the supplementary materials, with the exception of the Null series, which is combined with the 'Unimodal Alternative, High π_0' series. Both the 'Null' and 'Unimodal Alternative, High π_0' series are also evaluated in the Polyester *in silico* experiments, and these results are provided as a separate separate file.

**Series**	**Non-null Effect Size Distribution**[11]	**Non-null Genes N(%)**[12]	**Covariate Strength**[10]	**Figures**	**Additional File**
Null	–	0	–	–	2
Unimodal Alternative, High π_0	Unimodal	2000 (≈30%)	Strong<br>Weak<br>Uninformative	S1, S15, S11, S3, S10	2
Unimodal Alternative, Low π_0	Unimodal	500 (≈7.5%)	Strong<br>Weak<br>Uninformative	S3, S10	3
Bimodal Alternative, High π_0	Bimodal	2000 (≈30%)	Strong<br>Weak<br>Uninformative	S3, S10	4
Bimodal Alternative, Low π_0	Bimodal	500 (≈7.5%)	Strong<br>Weak<br>Uninformative	S3, S10	5

[10]In all cases, non-null genes were selected using probability weights sampled from a logistic function (where weights $w(u) = \frac{1}{1+e^{-10u+5}}$, and $u \sim U(0,1)$). The strongly informative covariate X_s was equal to the logistic sampling weight w. The weakly informative covariate X_w was equal to the logisitic sampling weight plus noise: $w + \epsilon$, where $\epsilon \sim N(0, 0.25)$, truncated such that $X_w \in (0,1)$. The uninformative X_u covariate was unrelated to the sampling weights and drawn from a uniform distribution such that $X_u \sim U(0,1)$

[11]For unimodal alternative effect size distributions, the observed fold changes for the selected non-null genes in a non-null empirical comparison were used. For bimodal alternatives, observed test statistics z from an empirical non-null comparison were sampled with probability weights $w(z) = f(|x|; \alpha, \beta)$, where f is the Gamma probability density function (with shape and rate parameters $\alpha = 4.5$ and $\beta = 1 - 1e^{-4}$, respectively). The corresponding effect sizes (fold changes, FC) for ashq were calculated assuming a fixed standard error: $FC = z\sigma_m$, where σ_m is the median standard error of the log_2 fold change across all genes.

[12]Total number of genes considered (with mean expression across all samples greater than 1 raw count) is 6553. A small number of genes are removed from each replicate if DESeq2 does not return a p-value.

Table S3 Simulation settings. The results from each series of simulations is reported as a separate Additional file in the supplementary materials.

Series	M	Test Statistic Distribution	Effect Size Distribution	Marginal Null Proportion ($\bar{\pi}_0$)	Covariate Relationship ($p(x;\bar{\pi}_0)$)	Figures
Null	20000	$N(0,1)$<br>t_{11}<br>t_5<br>χ^2_4	–	1.00	–	–
Informative (cubic)	20000	$N(0,1)$<br>t_{11}<br>t_5<br>χ^2_4	$N(3,1)$<br>$N(3,1)$<br>$N(3,1)$<br>$N(15,1)$	0.90	$p^{\text{cubic}}(x;\bar{\pi}_0)$	Figure S6,Figure S5
Informative (step)	20000	$N(0,1)$<br>t_{11}<br>t_5<br>χ^2_4	$N(3,1)$<br>$N(3,1)$<br>$N(3,1)$<br>$N(15,1)$	0.90	$p^{\text{step}}(x;\bar{\pi}_0)$	Figure S5
Informative (sine)	20000	$N(0,1)$<br>t_{11}<br>t_5<br>χ^2_4	$N(3,1)$<br>$N(3,1)$<br>$N(3,1)$<br>$N(15,1)$	0.90	$p^{\text{sine}}(x;\bar{\pi}_0)$	Figure S5
Informative (cosine)	20000	$N(0,1)$<br>t_{11}<br>t_5<br>χ^2_4	$N(3,1)$<br>$N(3,1)$<br>$N(3,1)$<br>$N(15,1)$	0.90	$p^{\text{cosine}}(x;\bar{\pi}_0)$	Figure S5
Unimodal Effect Sizes	20000	$N(0,1)$	bimodal<br>spiky<br>flat-top<br>skewed	0.90	$p^{\text{cubic}}(x;\bar{\pi}_0)$	Figure S7
Unimodal Effect Sizes (t_{11} test statistics)	20000	t_{11}	bimodal<br>spiky<br>flat-top<br>skewed	0.90	$p^{\text{cubic}}(x;\bar{\pi}_0)$	–
Unimodal Effect Sizes (25% non-null)	20000	$N(0,1)$	bimodal<br>spiky<br>flat-top<br>skewed	0.75	$p^{\text{cubic}}(x;\bar{\pi}_0)$	Figure S8
Varying M Tests	100<br>500<br>1000<br>5000<br>10000<br>50000	$N(0,1)$	$N(3,1)$	0.90	$p^{\text{sine}}(x;\bar{\pi}_0)$	Figure S4C-D
Varying Null Proportion	20000	$N(0,1)$	$N(2,1)$	0.05<br>0.10<br>...<br>0.95<br>0.99	$p^{\text{sine}}(x;\bar{\pi}_0)$	Figure S4E-F
Varying Null Proportion (t_{11} test statistics)	20000	$N(0,1)$	$N(2,1)$	0.05<br>0.10<br>...<br>0.95<br>0.99	$p^{\text{sine}}(x;\bar{\pi}_0)$	–
Varying Informativeness (continuous $p(x;\delta)$)	20000	$N(0,1)$	$N(2,1)$	0.80	$p^{\text{c-info}}(x;\delta=0)$<br>$p^{\text{c-info}}(x;\delta=5)$<br>...<br>$p^{\text{c-info}}(x;\delta=100)$	Figure S4A-B
Varying Informativeness (discrete $p(x;\delta)$)	20000	$N(0,1)$	$N(2,1)$	0.80	$p^{\text{d-info}}(x;\delta=0)$<br>$p^{\text{d-info}}(x;\delta=5)$<br>...<br>$p^{\text{d-info}}(x;\delta=100)$	–

Table S4 Case Study results. For each case study and method, mean proportion of maximum number of rejections by any method at $\alpha = 0.05$, as shown in the left panel of Figure S15. Range is given in parentheses. A '-' indicates that the method was not applied to the specified case studies.

Method	GWAS	RNA-seq	ChIP-seq	GSEA	Microbiome	scRNA-seq
ashq	0.9 (0.79-1)	0.73 (0.47-1)	-	-	-	-
fdrreg-t	0.69 (0.66-0.72)	0.48 (0.42-0.55)	-	-	-	-
fdrreg-e	0.78 (0.71-0.84)	0.52 (0.04-1)	-	-	-	-
lfdr	0.96 (0.91-1)	0.57 (0.56-0.57)	1 (1-1)	0.98 (0.95-1)	0.98 (0.96-1)	1 (0.96-1)
adapt-glm	0.34 (0-0.69)	0.47 (0.38-0.56)	0.58 (0.19-0.93)	0.5 (0-1)	0.41 (0-1)	0.87 (0.61-1)
bl	0.64 (0.57-0.7)	0.45 (0.4-0.49)	0.64 (0.28-0.92)	0.65 (0.51-0.79)	0.78 (0.22-1)	0.88 (0.63-1)
qvalue	0.63 (0.55-0.7)	0.44 (0.4-0.48)	0.61 (0.28-0.92)	0.61 (0.48-0.75)	0.7 (0.06-1)	0.84 (0.59-1)
ihw	0.79 (0.73-0.84)	0.45 (0.41-0.48)	0.48 (0.12-0.82)	0.63 (0.61-0.65)	0.65 (0.34-0.92)	0.68 (0.5-0.78)
bh	0.62 (0.54-0.69)	0.39 (0.38-0.4)	0.44 (0.06-0.82)	0.5 (0.44-0.55)	0.53 (0.04-0.92)	0.64 (0.45-0.77)
bonf	0.17 (0.15-0.18)	0.08 (0.04-0.11)	0.16 (0-0.32)	0 (0-0)	0.16 (0-0.46)	0.16 (0.07-0.23)

1 Supplementary *in silico* experiment results

Here we summarize the benchmarking results of fdrreg-e, which was excluded from the main results due to its unstable and often inferior performance compared to its counterpart fdrreg-t. The difference between these two implementations of FDRreg is that fdrreg-t assumes the null distribution of test statistics is standard normal, while fdrreg-e estimates the null distribution of test statistics empirically. We find this estimation procedure to be sensitive to settings of the distribution of effect sizes and proportion of non-null tests in particular.

1.1 Summary of fdrreg-e performance

We found that while modern FDR methods generally led to a higher true positive rate (TPR), or power, in the *in silico* experiments and simulations, fdrreg-e was sometimes as conservative as the Bonferroni correction (Figure S3). The increase in TPR of fdrreg-e sometimes showed substantial improvement over modern methods in several simulation settings (Figures S3, S4, S5, S6, S7, S8). However, these gains were often accompanied by a lack of FDR control, highlighting the sensitivity of fdrreg-e to underlying model assumptions.

1.1.1 Number of tests

We observed that the FDR control of fdrreg-e was sensitive to the number of tests in simulation. Specifically, FDR was substantially inflated when fdrreg-e was applied to fewer than 1,000 tests (Figure S4C). FDR control generally improved as the number of tests increased.

1.1.2 Proportion of non-null tests

The performance of fdrreg-e was particularly sensitive to extreme changes in the proportion of non-null tests. In simulations, fdrreg-e exhibited inflated FDR when the proportion of non-null hypotheses was near 50% (Figure S4E), and suffered from low TPR when there were more than 20% non-null hypotheses, excluding settings where the FDR was not controlled (Figure S4F). In the yeast *in silico* experiments, we also observed that fdrreg-e was more conservative when the proportion of non-null genes was 30% compared to when it was 7.5% (Figure S3). Similar results were also observed in a series of simulations where unimodal effect sizes were used when the proportion of non-null tests was increased from 10% (Figure S7) to 25% (Figure S8).

1.1.3 Distribution of test statistics

We observed that the performance of fdrreg-e declined when the normality assumption of the test statistic was violated (Figure S6B-C). FDR was considerably inflated when it was applied to t-distributed test statistics. As expected, the increase in FDR was greater for the heavier-tailed t distribution with fewer degrees of freedom (Figure S6B).

1.1.4 Distribution of effect sizes

In addition to distributional assumptions on the test statistic, empirical FDRreg requires distributional assumptions on the effect size. Specifically, the empirical null framework used in fdrreg-e relies on [14] to estimate the distribution of null test statistics which requires that all test statistics with values near zero are null, referred to as the 'zero assumption'. If this is not true, as is the case when the effect sizes are unimodal, the estimation of the null distribution is unidentifiable and may become overly wide, resulting in conservative behavior.

To investigate the sensitivity of these methods to the distribution of effect sizes, multiple distributions of the effect sizes were considered in both yeast *in silico* experiments and simulations. Both unimodal effect size distributions and those following the assumption of fdrreg-e, with most non-null effects greater than zero (Figure S6A), were considered. While most simulations included the latter, simulations were also performed with a set of unimodal effect size distributions described in [2] (Figure S7 and S8). In the yeast *in silico* experiments, two conditions were investigated - a unimodal and a bimodal case.

As expected, we observed that when the zero assumption of empirical FDRreg is violated, fdrreg-e was more conservative in both the yeast *in silico* experiments (Figure S3) and in simulation (Figures S7 and S8).

We also note that while it is simple to check distributional assumptions on the overall distribution of test statistics or effect sizes, in practice it is impossible to check the distributional assumptions of empirical FDRreg under the alternative, since they rely on knowing which tests are non-null.

2 Supplementary case study results

To illustrate what types of covariates may be informative in controlling the FDR in different computational biology contexts, we compared the methods using six case studies including genome-wide association testing (Section 2.1), gene set analysis (Section 2.2), detecting differentially expressed genes in bulk RNA-seq (Section 2.3) and single-cell RNA-seq (Section 2.4), differential binding in ChIP-seq (Section 2.5), and differential abundance testing in the microbiome (Section 2.6). Here we provide additional results for each case study to complement the summary provided in the main text. For full details of the analyses and results, refer to Additional files 21-41.

2.1 Case-study: Genome-Wide Association Studies

Genome-Wide Association Studies (GWAS) are typically carried out on large cohorts of independent subjects in order to test for association of individual genetic variants with a phenotype. The genetic variants are generally measured using microarrays containing probes for up to several million Single Nucleotide Polymorphisms (SNPs). These SNP probes target single base-pair DNA sites that have been shown to vary across a population. To boost power, meta-analyses of GWAS group together many studies, commonly including hundreds of thousands to millions of SNPs, with heterogeneous effect sizes and a wide range of sample size at each loci.

We analyzed a GWAS experiment that carried out a meta-analysis of hundreds of thousands of individuals for more than two million SNPs for association of genetic variants with Body Mass Index (BMI) [15]. As informative covariates, we considered (1) the minor allele frequency (MAF), or the proportion of the population which exhibits the less common allele, and (2) the number of samples for which each SNP was tested for association in the corresponding meta-analysis. In total, 196,969 approximately independent SNPs (out of 2,456,142) were included in the FDR analysis.

For each covariate, we examined whether its rank was associated with the p-value distribution. As expected, larger values of the sample size resulted in an enrichment for smaller p-values. Additionally, intermediate values of the MAF were associated with an enrichment for smaller p-values. This is expected since an MAF near 0.5 balances the number of samples with each allele, thereby maximizing power to detect a difference. For both covariates, the distribution of moderate to large p-values appeared uniform and independent of the value of the covariate. For methods that include a covariate, similar numbers of SNPs were rejected at the 0.05 level for either covariate.

For both informative covariates, we found lfdr, ihw, and fdr-e rejected the largest number of hypotheses, followed by fdr-t. The sample size covariate appeared to be more informative than MAF for lfdr and ihw, as both methods rejected more than ashq, whereas ashq found more discoveries than all covariate-aware methods that used MAF. Neither covariate seemed to be very informative for bl, as it did not have much gain over bh or qvalue. adapt-glm was more conservative than Bonferroni with the MAF covariate, but was ranked above bl using the sample size covariate. The overlap among the methods was high, with the largest set sizes containing SNPs rejected by all methods except Bonferonni and/or adapt-glm for both covariate comparisons. The next largest set size was the SNPs rejected by ashq exclusively.

2.2 Case-study: Gene set analyses

Gene set analysis is commonly used to provide insights to results of differential expression analysis. These methods aim to identify gene sets such as Gene Ontology (GO) categories or biological pathways that exhibit a pattern of differential expression. One class of methods, called overrepresentation approaches, test each gene set for a higher number of differentially expressed genes than expected by chance [16]. Another class of methods, called functional class scoring approaches, test each gene set for a coordinated change in expression [16]. While the former operates on a list of differentially expressed genes and does not consider the magnitude or direction of effect, the latter uses information from all genes, and can even detect small coordinated changes across many genes that are not significantly DE individually. We investigated the use of an informative covariate in GOseq [17], an overrepresentation test, as well as Gene Set Enrichment Analysis (GSEA) [18], a functional class scoring approach. Since the sizes of gene sets differ substantially and these size differences translate into differences in power, we hypothesized that multiple-testing correction in gene set analysis would benefit from methods that incorporate information about set sizes.

We used two RNA-seq datasets that investigated gene expression changes (1) between cerebellum and cerebral cortex [19] and (2) upon differentiation of hematopoietic stem cells (HSCs) into multipotent progenitors (MPP) [20]. We obtained 9,853 and 1,336 differentially expressed genes with FDR below 0.10 (using BH) for the human

and mouse datasets, respectively. We observed that for both GSEA and GOseq larger gene sets were more likely to have smaller p-values than smaller gene sets. Thus, the covariate was informative. In addition, the covariate appeared to be independent under the null hypothesis for GSEA, as evaluated by the histogram of p-values stratified by gene set size bins. However, upon evaluation of the stratified histograms of GOseq p-values, we observed that the distribution of p-values in the larger range was quite different for different covariate bins. This suggests that gene set size is not independent under the null hypothesis for GOseq, so the assumptions of methods which use an independent covariate are violated. Thus, we do not include the GOseq method in the benchmarking study and instead proceed with GSEA p-values only.

In this case study, we excluded the methods fdrreg-e, fdrreg-t, and ashq since they require standard errors and test statistics that GSEA does not provide. Overall lfdr, bl, and ihw rejected more hypotheses compared to the other methods. However, the ranking among these methods was not the same between the different datasets. Fort the mouse dataset, lfdr found the most rejections at smaller α levels (less than 0.05), but adapt-glm found the most at higher α levels. This was followed by BL, qvalue, and then IHW. For the human dataset, lfdr found the most rejections at all α levels, followed by IHW and then BL, and adapt-glm was more conservative than BH for almost all α levels. As expected, performance using the random (uninformative) covariate of BL and IHW was almost identical to qvalue and BH, respectively. However, the adapt-glm using the uninformative covariate was quite different in the two datasets, with no rejections in the human, and more rejections than any other method in the mouse (at $\alpha > 0.05$).

2.3 Case-study: Differential gene expression in bulk RNA-seq

High-throughput sequencing of mRNA molecules has become the standard for transcriptome profiling. A central analysis task is to determine which genes are deferentially expressed between two biological conditions. Statistical models have been established to address this question including DESeq2 [21] and edgeR [22]. These methods return per gene p-values that are further adjusted for multiple testing, typically using the Benjamini-Hochberg procedure.

We assessed the performance of modern FDR methods in the context of differential expression on two RNA-seq datasets. The first dataset consisted of two tissues of 10 individuals from the *GTEx* project and the second dataset consisted of a mouse knockdown experiment of the microRNA *mir200c*. For FDR methods that can use an informative covariate, we used mean expression across samples. We confirmed that this covariate was indeed informative for both datasets.

For the *GTEx* dataset, ashq found more rejections than any other method. At a FDR of 10%, the number of rejections of ashq was more than twice the number of rejections from any of rest of the methods, and the largest gene set was the set of genes found by ashq and no other methods. Following ashq, lfdr, adapt-glm, and fdrreg-t performed similarly. bl found almost the same number of rejections as qvalue, and ihw found slightly more than bh. fdrreg-e was as conservative as Bonferroni. The ranking of the methods based on the number of rejections was consistent across different strata of the covariate.

For the *mir200c* dataset the ranking of the methods was very different compared to the *GTEx* dataset. Here, fdrreg-e found the most rejections by far, and the largest gene set was the set of genes found by fdrreg-e and no other methods. The next highest ranking methods were lfdr, ihw, and ashq, followed by bl, qval, bh, fdrreg-t, and adapt-glm which all performed similarly. For this dataset, the ranking of methods changed substantially across strata of the covariate. For example, among the hypothesis falling between the 50th and 75th percentile of the covariate, lfdr was ranked second (the next highest ranked method after fdrreg-e) but among the hypothesis between the 75th and 100th percentile of the covariate, ashq was ranked second.

2.4 Case-study: Differential gene expression in single-cell RNA-seq

Over the past 5 years, breakthroughs in microfluidics and droplet-based RNA capture technologies have made it possible to sequence the transcriptome of individual cells rather than populations of cells. Quantification of single-cell RNA-seq (scRNA-seq) reads results in a matrix of counts by cells for each sample. The primary applications of scRNA-seq have been in describing cellular heterogeneity in primary tissues, differences in cellular heterogeneity in disease, and discovery of novel cell subpopulations. Differential gene expression of scRNA-seq is used to determine gene sets which distinguish cell populations within the same biological condition, and between cell populations in different samples or conditions.

In this case-study, we examined differences in gene expression in two different biological systems. First, we detected differentially expressed genes between neoplastic glioblastoma cells sampled from a patient's tumor core

with those sampled from nearby peripheral tissue [23]. In addition, we also detected differentially expressed genes between murine macrophage cells that were stimulated to produce an immune response with an unstimulated population [24]. We carried out differential expression analyses using two different methods developed for scRNA-seq: scDD [25] and MAST [26], as well as the Wilcoxon Rank-Sum test.

We examined the mean nonzero expression and detection rate (defined as the proportion of cells expressing a given gene) as potentially informative covariates. For both datasets and all three differential expression methods, we found that mean nonzero expression and detection rate were both informative and approximately independent under the null hypothesis, satisfying the conditions for suitability of inclusion as an informative covariate for controlling FDR. All methods returned more rejections of genes with high nonzero mean and detection rate. They also tended to slightly favor genes with extremely low detection rate.

Across datasets, covariates, and differential expression tests, lfdr usually found the most rejections, followed by bl and adapt-glm. However, at smaller α values, adapt-glm was one of the most conservative methods. The ihw and qvalue methods were next, with their rank dependent the dataset and differential expression test used. While a gain in rejections for ihw over bh was apparent in the human dataset, the performance of ihw was very similar to bh in the mouse dataset.

2.5 Case-study: Differential binding in ChIP-seq

ChIP-seq has been widely used to detect protein binding regions and histone modifications in DNA. Testing difference of ChIP-seq signals between conditions usually contains two steps: firstly, defining sets of regions for which the ChIP-seq coverage are quantified; secondly, comparing quantified coverages for testing the statistical significance of differential binding regions. In the first step, regions can be defined by peak calling from samples, based on their signal in sliding windows [27], or by *a priori* interest. In this study we benchmarked results from the latter two approaches by analyzing H3K4me3 data from two widely studied cell lines. Because H3K4me3 is an active marker of gene expression, its signal is most active in promoter regions. This allowed us to pursue an analysis of promoters as regions of interest. We also benchmark the results using the sliding window approach csaw to define *de novo* regions on the H3K4me3 dataset as well as an additional dataset comparing CREB protein binding (CRB) in wild-type versus knock-out mice. We exclude ashq and FDRreg methods from the sliding window analyses since csaw does not provide a standard error or test statistic.

Based on observations that differentially bound peaks tend to have higher coverage, we investigated the use of mean coverage as an informative covariate. In the promoter analysis, the p-value histograms showed that high coverage groups are more highly enriched for significant p-values different, suggesting mean coverage is an informative covariate. Likewise, we observed that wider windows in the *de novo* analysis tend to have more significant p-values. The distribution of p-values under the null in both cases appeared approximately uniform.

In the promoter analysis, ashq detected the highest number of differential binding regions by far, followed by lfdr, fdrreg-t, bl, qvalue, and adapt-glm, which all performed similarly. In the sliding window analyses, lfdr rejected the most hypotheses in both datasets, followed by adapt-glm, bl, and qvalue, which performed similarly to one another. In both datasets, the next lowest methods were ihw and bh, where the advantage of ihw only observed in the CBP csaw analysis. Finally, fdrreg-e was more conservative than Bonferroni in the promoter analysis.

2.6 Case-study: Differential abundance testing and correlations in microbiome data analysis

16S rRNA sequencing provides an overview of the microbial community in a given sample, and is a common and accessible way to identify relationships between microbial communities and phenotypes of interest. For example, differential abundance testing is often used to identify bacterial taxa which are enriched or depleted in a disease state, and non-parametric correlations between taxa abundances and phenotypes can be calculated when phenotypes of interest are continuous (e.g. body mass index). However, 16S rRNA datasets are high-dimensional, noisy, and sparse, and biological effects can be weak, complicating many statistical analyses and limiting power to detect true associations [28, 29]. Furthermore, environmental samples tend to have many thousands of taxa, which further complicates our ability to identify significant associations

We performed differential abundance tests on the OTU and genus levels for three different datasets from the microbiomeHD database: (1) obesity, where we do not expect a large disease-associated signal [28, 30], (2) inflammatory bowel disease (IBD), which seems to have an intermediate number of disease-associated bacteria [31, 32], and (3) infectious diarrhea (Clostridium difficile (CDI) and non-CDI), where the disease-associated signal is very strong [32, 33]. We also performed Spearman correlation tests between OTU relative abundances

and the respective values of three geochemical variables, measured from wells from a contaminated former S-3 waste disposal site in the Bear Creek watershed in Oak Ridge, Tennessee, part of the Department of Energy's Oak Ridge Field Research Center [34]. The geochemical variables were chosen based on their ability to be predicted by the microbial community in [34]: pH, Al, and SO_4, where we expect strong, intermediate, and weak associations with the microbial abundances, respectively.

We examined the ubiquity (defined as the proportion of samples with non-zero abundance of each taxa) and mean non-zero abundance of taxa as potentially informative covariates. We found that ubiquity was informative and approximately independent under the null hypothesis, satisfying the conditions for suitability of inclusion as an informative covariate for controlling FDR. Mean non-zero abundance appeared less informative than ubiquity, as it typically showed a less striking pattern in diagnostic plots of p-values by covariate value.

OTU-level differential abundance analyses did not have sufficient power to detect any significant differences in the IBD, CRC, and obesity datasets. Similarly, no OTUs correlated with SO_4 levels and ubiquity was not informative in this case. In addition, very few rejections were found in the CRC dataset at the genus level. Consequently, ubiquity was not informative in these "null" analyses and almost all FDR-correction methods found no significant associations. These "null" results are excluded from the results in the main text.

For the other analyses (genus-level differential abundance, OTU-level differential abundance in diarrhea, OTU-level correlation analyses for pH and Al), ubiquity was informative and the FDR-correction methods which incorporated this information tended to recover more significant associations than naive methods. When there were enough tests for it to be applied, lfdr typically found the most rejections. This was usually followed by bl and qvalue, with the gain of bl over qvalue variable by dataset. In the correlation analyses, however, ihw found more rejections than bl and qvalue. The performance of adapt-glm was usually highly variable, both within a dataset and across datasets: it either had a very different ranking at different α levels (obesity), found the among the most rejections (correlation of pH), or found no rejections at all (IBD, CRC). In cases with very few tests (e.g. genus-level analyses), ihw used only 1 covariate bin and reduced to bh as expected.

References

1. Soneson C, Robinson MD. iCOBRA: open, reproducible, standardized and live method benchmarking. Nature Methods. 2016;13(4):283.
2. Stephens M. False discovery rates: a new deal. Biostatistics. 2016;18:275–294.
3. Dunn OJ. Multiple comparisons among means. Journal of the American Statistical Association. 1961;56(293):52–64.
4. Bonferroni CE. Teoria statistica delle classi e calcolo delle probabilità. Pubblicazioni del R Istituto Superiore di Scienze Economiche e Commerciali di Firenze. 1936;8:3–62.
5. Benjamini Y, Hochberg Y. Controlling the false discovery rate: a practical and powerful approach to multiple testing. Journal of the Royal Statistical Society Series B. 1995;p. 289–300.
6. Ignatiadis N, Klaus B, Zaugg JB, Huber W. Data-driven hypothesis weighting increases detection power in genome-scale multiple testing. Nature Methods. 2016;13:577–580.
7. Storey JD. A direct approach to estimating false discovery. Journal of the Royal Statistical Society Series B). 2002;64(3):479–498.
8. Boca SM, Leek JT. A direct approach to estimating false discovery rates conditional on covariates. bioRxiv. 2017;Available from: https://doi.org/10.1101/035675.
9. Cai TT, Sun W. Simultaneous testing of grouped hypotheses: Finding needles in multiple haystacks. Journal of the American Statistical Association. 2009;104:1467–1481.
10. Lei L, Fithian W. AdaPT: an interactive procedure for multiple testing with side information. Journal of the Royal Statistical Society: Series B. 2018;80:649–679.
11. Barber RF, Candès EJ, et al. Controlling the false discovery rate via knockoffs. The Annals of Statistics. 2015;43(5):2055–2085.
12. Arias-Castro E, Chen S, et al. Distribution-free multiple testing. Electronic Journal of Statistics. 2017;11(1):1983–2001.
13. Scott JG, Kelly RC, Smith MA, Zhou P, Kass RE. False discovery rate regression: an application to neural synchrony detection in primary visual cortex. Journal of the American Statistical Association. 2015;110:459–471.
14. Efron B. Microarrays, Empirical Bayes and the Two-Groups Model. Statistical Science. 2008;23(1):1–22.
15. Speliotes EK, Willer CJ, Berndt KL S I Monda, Thorleifsson G, Jackson AU, Allen CM H L Lindgren, et al. Association analyses of 249,796 individuals reveal 18 new loci associated with body mass index. Nature Genetics. 2010;42(11):937–948.
16. Mathur R, Rotroff D, Ma J, Shojaie A, Motsinger-Reif A. Gene set analysis methods: a systematic comparison. BioData Mining. 2018;11(1):8.
17. Young MD, Wakefield MJ, Smyth GK, Oshlack A. Gene ontology analysis for RNA-seq: accounting for selection bias. Genome Biology. 2010;11(2):R14. Available from: http://dx.doi.org/10.1186/gb-2010-11-2-r14.
18. Sergushichev A. An algorithm for fast preranked gene set enrichment analysis using cumulative statistic calculation. BioRxiv. 2016;Available from: https://doi.org/10.1101/060012.
19. Mele M, Ferreira PG, Reverter F, DeLuca DS, Monlong J, Sammeth M, et al. The human transcriptome across tissues and individuals. Science. 2015 May;348(6235):660665. Available from: http://dx.doi.org/10.1126/science.aaa0355.
20. Cabezas-Wallscheid N, Klimmeck D, Hansson J, Lipka D, Reyes A, Wang Q, et al. Identification of Regulatory Networks in HSCs and Their Immediate Progeny via Integrated Proteome, Transcriptome, and DNA Methylome Analysis. Cell Stem Cell. 2014 Oct;15(4):507522. Available from: http://dx.doi.org/10.1016/j.stem.2014.07.005.
21. Love MI, Huber W, Anders S. Moderated estimation of fold change and dispersion for RNA-seq data with DESeq2. Genome Biology. 2014;15(12):550.
22. Robinson MD, McCarthy DJ, Smyth GK. edgeR: a Bioconductor package for differential expression analysis of digital gene expression data. Bioinformatics. 2009 Nov;26(1):139140. Available from: http://dx.doi.org/10.1093/bioinformatics/btp616.
23. Darmanis S, Sloan SA, Croote D, Mignardi M, Chernikova S, Samghababi P, et al. Single-Cell RNA-Seq Analysis of Infiltrating Neoplastic Cells at the Migrating Front of Human Glioblastoma. Cell Reports. 2017;21(5):1399–1410.

24. Lane K, Van Valen D, DeFelice MM, Macklin DN, Kudo T, Jaimovich A, et al. Measuring Signaling and RNA-Seq in the Same Cell Links Gene Expression to Dynamic Patterns of NF-κB Activation. Cell Systems. 2017;4(4):458–469.
25. Korthauer KD, Chu LF, Newton MA, Li Y, Thomson J, Stewart R, et al. A statistical approach for identifying differential distributions in single-cell RNA-seq experiments. Genome Biology. 2016;17(1):222.
26. Finak G, McDavid A, Yajima M, Deng J, Gersuk V, Shalek AK, et al. MAST: a flexible statistical framework for assessing transcriptional changes and characterizing heterogeneity in single-cell RNA sequencing data. Genome Biology. 2015;16(1):278.
27. Lun AT, Smyth GK. csaw: a Bioconductor package for differential binding analysis of ChIP-seq data using sliding windows. Nucleic Acids Research. 2015;44(5):e45–e45.
28. Sze MA, Schloss PD. Looking for a Signal in the Noise: Revisiting Obesity and the Microbiome. mBio. 2016;7(4):e01018–16.
29. Paulson JN, Stine OC, Bravo HC, Pop M. Differential abundance analysis for microbial marker-gene surveys. Nature Methods. 2013;10(12):1200–1202.
30. Goodrich JK, Waters JL, Poole AC, Sutter JL, Koren O, Blekhman R, et al. Human Genetics Shape the Gut Microbiome. Cell. 2014;159(4):789–799.
31. Papa E, Docktor M, Smillie C, Weber S, Preheim SP, Gevers D, et al. Non-Invasive Mapping of the Gastrointestinal Microbiota Identifies Children with Inflammatory Bowel Disease. PLoS ONE. 2012;7(6):e39242.
32. Duvallet C, Gibbons SM, Gurry T, Irizarry RA, Alm EJ. Meta-analysis of gut microbiome studies identifies disease-specific and shared responses. Nature Communications. 2017;8(1).
33. Schubert AM, Rogers MAM, Ring C, Mogle J, Petrosino JP, Young VB, et al. Microbiome Data Distinguish Patients with Clostridium difficile Infection and Non-C. difficile-Associated Diarrhea from Healthy Controls. mBio. 2014;5(3):e01021–14–e01021–14.
34. Smith MB, Rocha AM, Smillie CS, Olesen SW, Paradis C, Wu L, et al. Natural Bacterial Communities Serve as Quantitative Geochemical Biosensors. mBio. 2015;6(3):e00326–15.
